# Coordination‐Responsive Longitudinal Relaxation Tuning as a Versatile MRI Sensing Protocol for Malignancy Targets

**DOI:** 10.1002/advs.201800021

**Published:** 2018-07-10

**Authors:** Kun Zhang, Yu Cheng, Weiwei Ren, Liping Sun, Chang Liu, Dan Wang, Lehang Guo, Huixiong Xu, Yongxiang Zhao

**Affiliations:** ^1^ Department of Medical Ultrasound Shanghai Tenth People's Hospital and Ultrasound Research and Education Institute Tongji University School of Medicine 301 Yan‐chang‐zhong Road Shanghai 200072 P. R. China; ^2^ National Center for International Research of Bio‐targeting Theranostics Guangxi Key Laboratory of Bio‐targeting Theranostics Collaborative Innovation Center for Tumor‐targeting Theranostics Guangxi Medical University 22 Shuang Yong Road Nanning Guangxi 530021 P. R. China; ^3^ Department of Imaging and Interventional Radiology Zhongshan‐Xuhui Hospital of Fudan University 966 Huai‐hai‐zhong Road Shanghai 200031 P. R. China

**Keywords:** biological targets, coordination‐dependent longitudinal relaxation tuning, pore diameters, spatial structures, T1‐weighted MRI

## Abstract

Biomarkers (e.g., acidity, H_2_O_2_, hypoxia, and specific molecules) as one primary component of tumor microenvironments are closely associated with occurrence, invasion, and metastasis of malignancy, thus can act as biological targets. However, their monitoring remains a challenging task. Herein, a coordination‐dependent longitudinal relaxation tuning (CLRT) that occurs between a Mn^2+^ “donor” and a Mn^2+^ “acceptor” is established to enable biological target sensing. Relying on the differences of coordination ability and spatial structure between donors and acceptors, the biological targets as Mn^2+^ acceptor can take Mn^2+^ away from the donors (i.e., modified ligands) in nanoscale probes, which consequently varies T1‐weighted (T1W) magnetic resonance imaging (MRI) signal. The coordination ability and spatial structure of the modified Mn^2+^ “donor” and the pore diameter of donor carrier are demonstrated to determine the feasibility, specificity, and generality of CLRT. With CLRT, this MRI‐based ruler is demonstrated for the successful specific detection of biological targets (i.e., hyaluronic acid and glutathione) of malignancy, and its potential in quantitative measurement of hyaluronic acid is further demonstrated. CLRT can serve as a novel and general sensing principle to augment the exploration of a wide range of biological systems.

## 1 Introduction

It has been extensively documented that tumor microenvironments (TMEs) play a predominant role in development, invasion, and metastasis of malignancy.^1^ A comprehensive understanding of TME is a prerequisite of establishing high‐efficient treatment approaches.^2^ Therefore, monitoring TMEs' biomarkers using many clinically noninvasive medical imaging technologies, e.g., ultrasound (US), photoacoustic (PA), magnetic resonance imaging (MRI), has aroused considerable attention with the progress of bio‐nanotechnology.^3^ In an attempt to explore biomarker sensing and tumor grading,^4^ a large variety of imaging principles have been developed to realize specific detection of some certain biomarker,^5^ whereas the versatility of these principles is poor. Typically, the ruler of fenton or fenton‐like principle was only confined to H_2_O_2_ sensing,[[qv: 5b]] and the principle of pH‐responsive Mn^2+^ dissolution‐enhanced T1 MRI signals is limited to pH sensing.^6^ New sensing protocol featuring high specificity and maximum versatility is still desirable but challenging.

Coordination‐responsive phenomena such as ligands exchange‐mediated hydrophobicity–hydrophilicity transition have been a reliable tool in biomedical research and gains increasing interest in scientific community.^7^ As a paradigm, ligands exchange resulting from competitive coordination can augment the application window via switching hydrophobic nanoparticles into hydrophilic ones preferably applicable for biological applications.[[qv: 7a]] In this study, a coordination‐responsive magnetic resonance tuning phenomenon, i.e., coordination‐responsive longitudinal relaxation tuning (CLRT), has been established to detect biological targets. CLRT originates from the competitive coordination, where the biological targets as Mn^2+^ acceptor seize Mn^2+^ from the donors (modified ligands) grafted in mesoporous carriers due to its stronger coordination ability toward Mn^2+^. Similar to distance‐dependent magnetic resonance tuning (MRET),^8^ this novel sensing principle is also basing on the variation of T1 magnetic resonance imaging (MRI) signals, since the more complex spatial structure of biological target can impair H_2_O longitudinal relaxation when the target is detected. Thus, CLRT also features a high versatility after rationally designing Mn^2+^ donor and mesoporous carriers aiming at a given biological target (designated as Mn^2+^ acceptor). This T1 modality was the preferable one in comparison to T2 modality in MRI.^8^

To explore its potential, two tailor‐made model systems was designed to detect two different tumor biological targets using the principle of CLRT, i.e., hyaluronic acid (HA) and glutathione (GSH), respectively.^9^ More significantly, we explored the determining factors of CLRT, and evaluated influences of carriers' pore diameter, coordination ability, and spatial structure of Mn^2+^ donor (modification ligands) on the specificity and feasibility of CLRT. Furthermore, the CLRT probe (i.e., HA) was employed to quantitatively detect HA in both in vitro and in vivo levels, consequently reflecting the potential of CLRT in quantitative analysis of various biomolecules in living systems.

## 2 Results and Discussion

### 2.1 CLRT Principle

**Figure 1:**
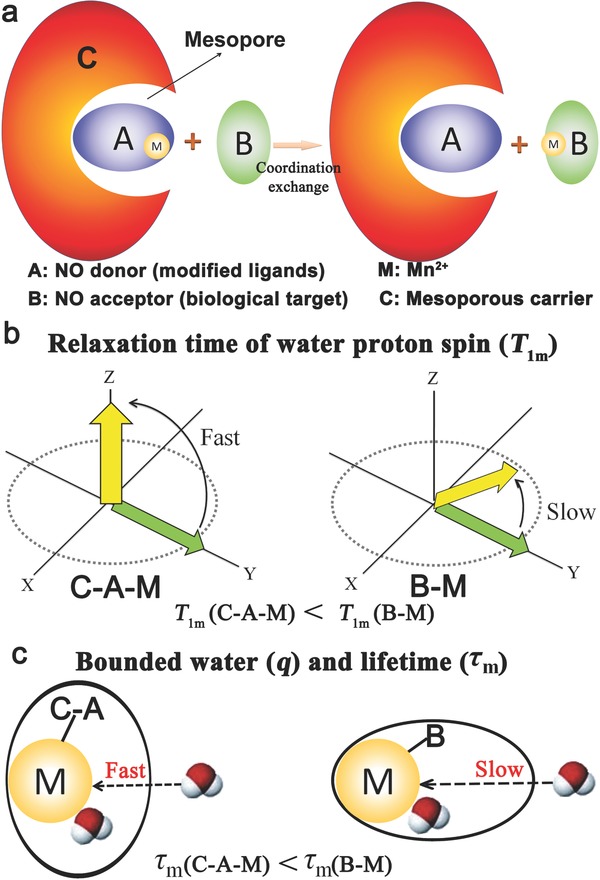
Principle of CLRT in detection of biological targets using T1W MRI sensing protocol. a) Principle of CLRT where A, B, C, and M represent Mn^2+^ donor (modified ligands), Mn^2+^ acceptor (biological target), mesoporous carriers, and Mn^2+^ ions, respectively, and B can capture M from C–A–M and generate B–M via coordination exchange. b) Relaxation time of water proton spin (*T*_1m_) in CLRT probe (C–A–M) and B chelator (B–M). c) Lifetime (τ_m_) of bounded water onto Mn^2+^ in C–A–M and B–M, which is determined by the exchange rate of H_2_O.

The CLRT principle involves three components, a modified ligands (Mn^2+^ donor, A), a biological target (Mn^2+^ acceptor, B), and a mesoporous carrier (C). Mesopore size of C and the differences of coordination ability and spatial structure between A and B determine the CLRT phenomenon (**Figure** **1**a). In detail, depending on its less size than the mesopore size of C and larger coordination ability than A, Mn^2+^ acceptor (B) is allowed to enter large mesopore of C, snatch Mn^2+^ from C–A–M (M presenting Mn^2+^) and generate B–M via the coordination exchange, realizing CLRT. The variation of longitudinal relaxation rate (1/*T*_1_) of Mn^2+^ can be employed to account for the CLRT, and 1/*T*_1_ is obtained according to the inner‐sphere relaxation formula (Equation (1))^10^

(1) $$\frac{1}{T_{1}}=\frac{qP_{m}}{T_{\mathrm{1m}}+\tau_{m}}$$

where *P*_m_ is the mole fraction of Mn^2+^, *T*_1m_ is the relaxation time of water proton spin, *q* is the number of bound water molecules per Mn^2+^ ion, τ_m_ is the lifetime of the solvent molecule in the complex (τ_m_ is the reciprocal of the solvent exchange rate, *k*_ex_). Among them, *T*_1m_, q, and τ_m_ are highlighted, because they influence the degree of CLRT phenomenon in this model (Figure 1a).

C–A can be regarded as a whole chelator of Mn^2+^, and it features larger spherical radius and higher rigidity than B chelator. Therefore, Mn^2+^ in B–M produced after coordination exchange shares shorter rotational correlation time (τ_R_) and longer *T*_1m_ than that in C–A–M, consequently slowing relaxation of water proton spin in B–M (Figure 1b), since larger molecule or size of Mn^2+^ chelator is beneficial for improving τ_R_ and decreasing *T*_1m_.^10^ This result eventually decreases longitudinal relaxation rate of Mn^2+^ (1/*T*_1_), resulting in a weaker T1‐weighted (T1W) MRI signal. With neglecting the limitation of C mesopore toward the water exchange, the more dense spatial structure of B than A also increases τ_m_ (Figure 1c), which further contributes to the decreased 1/*T*_1_ and T1W MRI. Therefore, the T1W can be expected to be tuned via the CLRT principle, and the biological target B can be detected when C–A is rationally designed. In particular, the decreased MRI signal as ruler via CLRT is more preferable than previous protocols, since it is difficult to discern whether the intensified MRI signal is resulted from particle accumulation or biological target triggering in previous protocols when using increased T1W MRI signal to reflect biological target.[[qv: 6a,8]]

### 2.2 CLRT Exploration in Hyaluronic Acid (HA) Detection

**Figure 2:**
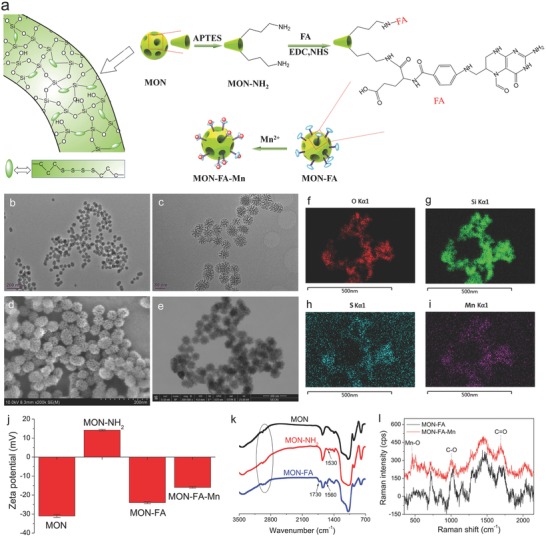
Syntheis and characterizations of CLRT probe (i.e., MON–FA–Mn). a) Preparation and composition schematic of MON–FA–Mn. b,c) TEM images of MON–FA–Mn. d) SEM and e) dark‐field STEM images of MON–FA–Mn. f–i) Atom mapping images of MON–FA–Mn including O, Si, S, Mn. j) Zeta potential of the intermediate products (e.g., MON, MON–NH_2_, and MON–FA) and the ultimate MON–FA–Mn. k) FTIR spectra of MON, MON–NH_2_, and MON–FA. l) Raman spectra of MON–FA and MON–FA–Mn.

The CLRT allows the variation of the T1 MRI signal to be utilized as a nanoscale ruler for detection of malignancy targets or biomarkers. To demonstrate its potential in detail, a model system with three components is designed, wherein C and A are mesoporous organosilica nanoparticles (MONs) (i.e., mesoporous carrier) and modified folic acid (FA) (i.e., Mn^2+^ donor), respectively, and a biomarker of malignancy, i.e., HA,[[qv: 9a,b]] is used as the Mn^2+^ acceptor (B). The C–A–M structure consisting of MON, FA, and Mn^2+^ (abbreviated into MON–FA–Mn) was obtained (**Figure** **2**a), wherein MONs platform composed of organic framework (R = –S–S–S–S–) were first prepared via a well‐established method,^11^ followed by –NH_2_ and FA modifications and Mn^2+^ chelation. The monodispersed MON–FA–Mn shares a maximum pore diameter of 13 nm (Figure 2; Figure S1, Supporting Information) that can allow most biological targets to enter mesopores and simultaneously ward off ultralarge metal transport proteins (MTPs). The chelated Mn^2+^ ions are uniformly distributed in mesoporous channels of MON platform (Figure 2f–i). The twice variations of zeta potential and two emerging stretching vibration peaks of –NH_2_ at 1530 cm^−1^ and C–O at 1730 cm^−1^ suggest the successful chelation of –NH_2_ and FA in sequence (Figure 2j,k). Accordingly, the average particle size gradually increases from 50 to 54 nm and 67 nm (MON–FA) as NH_2_ and FA modifications proceed in sequence (Figure S2, Supporting Information), and the modified amount of FA in MON–FA–Mn is 8.18% via TG analysis (Figure S3, Supporting Information). Depending on the hard acid‐hard base interaction,^12^ an emerging Raman characteristic peak of O–Mn at 459 cm^−1^ directly reflects the successful coordination between Mn^2+^ and FA (Figure 2l). Mn^2+^ chelation (6.8%) induces red shift of the C–O Raman peak from 1027 to 1005 cm^−1^, and zeta potential increase from −23.8 mV (MON–FA) to −16.1 mV. X‐ray photoelectron spectroscopy (XPS) spectrum of MON–FA–Mn indicates the valence of Mn in MON–FA–Mn is +2 (Figure S4, Supporting Information).

**Figure 3:**
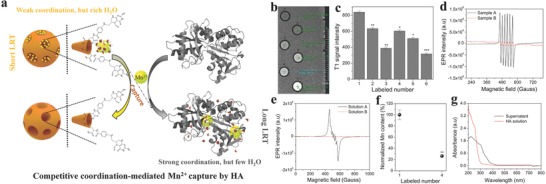
CLRT explorations via using MON–FA–Mn as the probe for HA detection. a) Operation schematic of CLRT for HA detection using MON–FA–Mn in which LRT represents longitudinal relaxation time. b,c) T1W MRI images (c) and d) corresponding T1W signal intensities of labeled number 1–6, wherein labeled number 1–6 represent MON–FA–Mn, the mixture of MON–FA–Mn and HA, HA solution, the centrifugal MON–FA–Mn after incubation with HA for 1.0 h, HA solution after incubation with MON–FA–Mn for 1.0 h, and water, respectively. d) ESR spectra of labeled number 1 (Sample A) and labeled number 4 (Sampled B). e) ESR spectra of labeled number 3 (Solution B) and labeled number 5 (Solution A). f) Normalized Mn content of labeled number 1 and 4 via ICP‐AES method. g) UV–vis spectra of labeled number 3 (HA solution) and labeled number 5 (supernatant). Note “*”, “**” and “***” represent *P* < 0.01, 0.005, and 0.001, respectively. Data are presented as the mean ± SEM.

The tumor biomarker associated with tumor metastasis,[[qv: 9b]] i.e., HA, was attempted to explore the potential of CLRT using its tailor‐made and specific model probe (MON–FA–Mn). The detailed process is indicated in **Figure** **3**a, wherein the biological target, i.e., HA (B) molecules, can capture Mn^2+^ from MON–FA–Mn (C–A–M) due to its stronger coordination than FA (A). This coordination exchange of Mn^2+^ from C–A–M to B–M alters the τ_R_, *T*_1m_, and τ_m_, because the more complex spatial structure of HA than FA intensify the stereo‐hindrance effect against H_2_O and the smaller size and poor rigid of HA chelator than MON–FA chelator reduce τ_R_.^10^ This phenomenon consequently results in a longitudinal relaxation variation that can be reflected by in vitro T1W MRI. It is clearly found that the T1W MRI performance (i.e., contrast and signal intensity) of MON–FA–Mn evidently drops when incubating with HA via comparing labeled number 1 and number 4 (Figure 3b,c), indicating Mn^2+^ capture by HA from MON–FA–Mn results in coordination‐responsive MRI. The electron spin resonance (ESR) spectra of MON–FA–Mn before and after incubating with HA and HA solution before and after incubating with MON–FA–Mn demonstrate Mn^2+^ ions departure from MON–FA–Mn (Figure 3d) and capture by HA (Figure 3e),^13^ consequently equipping HA solution with T1W MRI (Figure 3b,c) and resulting in an emerging UV–vis peak of HA–Mn at 310 nm in the UV–vis spectra (Figure 3g).^14^ Although Mn^2+^ entrapped in HA (labeled number 5) is more than that in labeled number 4 (74% vs 26%) (Figure 3f), the poorer T1W‐MRI outcome is observed. This result sufficiently demonstrates the reduced rigidity and radius of HA in comparison to MON–FA indeed diminish τ_R_ and increase *T*_1m_, and larger stereo‐hindrance in HA–Mn^2+^ severely hampers the exchange of H_2_O molecules bounded in HA–Mn and increases τ_m_.^10^ Therefore, T1W‐MRI performance of the incubated mixture of MON–FA–Mn and HA (labeled number 2) is lower than that in labeled number 1, demonstrating the potential of this CLRT.

We further examine the CLRT sensing concept in cellular‐level HA detection, and cell endocytosis via laser confocal scanning microscopy (LCSM) observation was also used to support CLRT. The fluorescence intensity of VX2 cells treated with FITC‐labeled MON–FA–Mn is found larger than that treated with MnCl_2_ and FITC‐labeled MON–FA–Mn in sequence (named as MnCl_2_ + MON–FA–Mn), as shown in Figure S5a in the Supporting Information, suggesting that Mn^2+^ captured by HA in VX cells can restore the targeting ability of FA. In contrast, the pretreatment of MnCl_2_ in MnCl_2_ + MON–FA–Mn first made HA molecules in VX2 cells coordinated with Mn^2+^, consequently avoiding capture of Mn^2+^ in MON–FA–Mn by HA and keeping the shielding effect of chelated Mn^2+^ toward FA targeting intact.^15^ Therefore, T1W MRI outcome of VX2 cells treated with MON–FA–Mn is poorer than that treated with MnCl_2_ + MON–FA–Mn (Figure S5b,c, Supporting Information). As well, VX2 cells were treated with MON–FA–Mn and the premixture of HA and MON–FA–Mn (HA + MON–FA–Mn), respectively. VX2 cells treated with HA + MON–FA–Mn exhibits stronger fluorescence intensity than those treated with MON–FA–Mn alone (Figure S6a, Supporting Information). The phenomenon indicates that the premixing of HA with MON–FA–Mn can permit HA to capture more Mn^2+^ from MON–FA–Mn, simultaneously liberate the shielding effect of chelated Mn^2+^ toward FA and allow more particles to enter VX2 cells via FA‐mediated active targeting. Therefore, more Mn^2+^ capture by HA in VX2 cells treated with HA + MON–FA–Mn results in longer τ_m_ and decreased T1W MRI (Figure S6b,c, Supporting Information) due to the larger stereo‐hindrance effect of HA that makes Mn^2+^ entrapped in HA inaccessible to H_2_O.

### 2.3 Determining Factors of CLRT

This observation of the coordination‐responsive T1W MRI signal is largely governed by the change in τ_R_, *T*_1m_, and τ_m_. Therefore, three concerns, i.e., pore diameter of mesoporous carrier (C), spatial structure, and coordination ability of modified ligands (Mn^2+^ donor, A), should be taken seriously when using CLRT as a MRI ruler for detection of biological targets, because they decide τ_R_, *T*_1m_, and τ_m_ of CLRT and further influence its feasibility, generality, and specificity. To comprehensively understand it, we also examine whether CLRT operates with other Mn^2+^ acceptors (e.g., GSH, fetal bovine serum (FBS), and ethylene diamine tetraacetic acid (EDTA)) using the HA‐specific model probe (MON–FA–Mn) and evaluate the influences of three concerns on CLRT involving feasibility and specificity.

Despite featuring low molecular weight, GSH fails to capture Mn^2+^ due to its weaker coordination ability toward Mn^2+^ than FA, which results in no evident variation of T1W MRI (Figure S7, Supporting Information). This phenomenon declares the coordination ability of initial modification ligands in probe manipulates the coordination‐responsive MRI. Especially for FBS, the maximum 13 nm mesopore in MON–FA–Mn prevented overlarge‐molecular‐weight (OMW) proteins from entering mesoporous channels, resulting in less than 7% Mn^2+^ release from MON–FA–Mn within 24 h (Figure S8, Supporting Information) and no evident variation of T1W MRI (Figure S9, Supporting Information). This phenomenon not only demonstrates the robust stability of MON–FA–Mn, but also suggests that the pore diameter of mesoporous carrier also governs the coordination‐responsive MRI.

As a universally accepted strong chelator toward metal ions,^16^ EDTA features stronger coordination ability than FA and lower molecular weight than HA, which determines EDTA can enter mesoporous channels of MON–FA–Mn and capture Mn^2+^. Depending on the decreased stereo‐hindrance of EDTA due to its simpler spatial structure than FA, EDTA can accelerate the exchange rate of water, resulting in shortened LRT and intensified T1W MRI (Figure S10, Supporting Information). Results indeed show Mn^2+^ captured by EDTA and intensified T1W MRI performance when MON–FA–Mn incubates with EDTA (Figures S11 and S12, Supporting Information), realizing the CLRT with a intensified T1W MRI ruler. Unfortunately, akin to previous strategies, it is also difficult to discern the intensified T1W MRI behavior is resulted from continuous accumulation of particles or CLRT.

### 2.4 Generalization Exploration of CLRT

Further generalization of CLRT with another class of mesoporous carriers (C), Mn^2+^ donors (modified agents, A), and Mn^2+^ acceptors (biological target, B) is examined. For instance, hollow mesoporous silica nanoparticles (HMSN) and –NH_2_‐contained side chains acted as carriers and Mn^2+^ donors, respectively, and the biological target of tumor, i.e., GSH,[[qv: 9c]] acts as the Mn^2+^ acceptor. Akin to MON–FA–Mn, the GSH‐specific HMSN–NH_2_–Mn was also obtained via a successive process, wherein HMSN platform with a diameter of 400 nm was first fabricated via our well‐established method,^17^ and then aminopropyltriethoxysilane (APTES) modification was carried out for grafting –NH_2_, followed by coordination with Mn^2+^ due to the excellent coordination ability of –NH_2_.^18^ The maximum pore diameter remains less than 3 nm (Figure S13, Supporting Information) and Mn^2+^ ions are also uniformly distributed in mesoporous channels (Figure S14, Supporting Information). The chelation amount of Mn^2+^ is around 4.5% via inductively coupled plasma‐atomic emission spectrometry (ICP‐AES) method.

**Figure 4:**
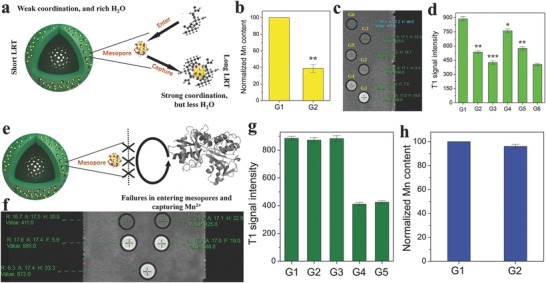
GSH detection using another model CLRT probe (HMSN–NH_2_–Mn). a) Operation schematic of CLRT for detecting GSH using HMSN–NH_2_–Mn in which LRT represents longitudinal relaxation time. b) Normalized Mn content of G1 and G2 via the ICP‐AES method. c) T1W MRI images of different groups (G1–G6), and d) the corresponding T1W signal intensity of G1–G6, where G1–G6 represent HMSN–NH_2_–Mn, the centrifugal HMSN–NH_2_–Mn after incubation with GSH, GSH solution, the mixture of HMSN–NH_2_–Mn and GSH, GSH solution after incubation with HMSN–NH_2_–Mn, and water, respectively. Note “*”, “**”, and “***” represent *P* <0.01, 0.005, and 0.001, respectively. e) The failure schematic of HMSN–NH_2_–Mn in detecting HA. f) Normalized Mn content of G1 and G2 via the ICP‐AES method, g) T1W MRI images of different groups (G1–G6), h) the corresponding T1W signal intensity of G1–G6, wherein G1–G6 represent HMSN–NH_2_–Mn, the cocentrifugal HMSN–NH_2_–Mn after incubation with HA, the mixture of HMSN–NH_2_–Mn and HA, HA solution after incubation with HMSN–NH_2_–Mn, and HA solution, respectively.

The 3 nm mesopores of HMSNs carriers and modification ligands determine the specific detection of GSH, e.g., the modification of –NH_2_‐carrying APTES indirectly determines the weaker coordination ability and reduced stereo‐hindrance of –NH_2_ than GSH, and the maximum 3 nm mesopore merely allows low‐molecular‐weight GSH to enter mesoporous channels and capture Mn^2+^, as depicted in **Figure** **4**a. Results definitely indicate Mn^2+^ in HMSN–NH_2_–Mn is captured by GSH via the competitive coordination (Figure 4b) and bring about a decreased MRI performance (Figure 4c,d). In contrast, the mesopore size (less than 3 nm) can prevent HA from entering mesoporous channels of HMSN–NH_2_–Mn and capturing Mn^2+^ (Figure 4h), ultimately resulting in no variation of T1W MRI (Figure 4f,g), as displayed in the schematic (Figure 4e). Therefore, CLRT can be considered as a general concept to provide coordination‐responsive MRI contrast effects that can be potentially utilized as a nanoscale ruler for specific detection of biological targets after rationally designing model system.

### 2.5 In Vitro and In Vivo CLRT Exploration for Quantitative HA Detection

**Figure 5:**
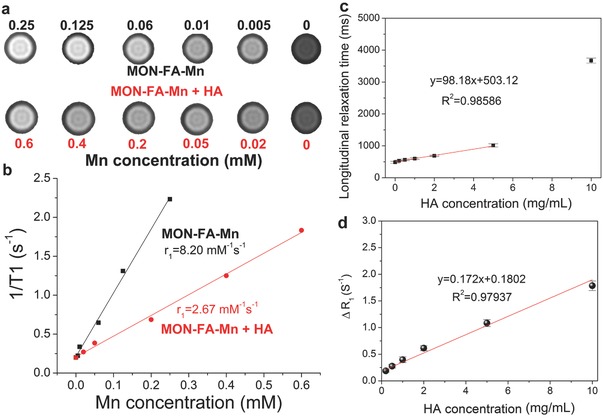
Variation of Longitudinal relaxation coefficient and time for HA detection. a) T1W MRI images of MON–FA–Mn dispersion and the mixture of MON–FA–Mn and HA (0.5 mg mL^−1^). b) *T*_1_^−1^ versus Mn molar concentrations for MON–FA–Mn and the mixture of MON–FA–Mn and HA (0.5 mg mL^−1^). c) Longitudinal relaxation time of the mixture consisting of MON–FA–Mn and HA with varied molar concentrations. d) correlation between HA concentration and ∆*R*_1_.

We further examine the CLRT sensing concept in in vitro and in vivo systems for HA detection. T﻿﻿_1_ relaxation coefficient (*r*1) of CLRT probe (MON–FA–Mn) before and after incubation with HA was further evaluated. Relying on the principle of CLRT, the *r*1 value of MON–FA–Mn considerably drops when mixing with HA (**Figure** **5**a,b), indicating the occurrence of Mn^2+^ captured by HA to MON–FA–Mn. An approximately linear relationship between longitudinal time and HA concentration is directly established within 5 × 10^−3^ m (Figure 5c). As a paradigm, the corresponding longitudinal time upon incubation with 0.3 mg mL^−1^ of HA is ≈513 ms according to the fitted liner equation, and this value is approximately identical to the actually measured value (511 ms), suggesting the detection limit of HA via CLRT using MON–FA–Mn as probe approaches 0.3 mg mL^−1^. More significantly, ∆*R*_1_, which is defined as the difference in longitudinal relaxation rate (*R*_1_ = 1/*T*_1_) between preincubation and postincubation with HA solution, shows a consistent decrease in the T1W MRI signal.^8^ Results manifest another linear correlation between HA concentration and ∆*R*_1_ is observed over the whole monitoring window, and more HA results in larger ∆*R*_1_ and decreased T1W MRI signal, as evidenced in Figure 5d. This linear correlation offers the possibility for the quantitative measurement of HA.

Encouraged by the successful results of in vitro HA detection, in vivo HA detection using the CLRT sensing ruler was implemented on VX2 tumor subcutaneously implanted in New Zealand rabbits. MON–FA–Mn particles share an excellent biocompatibility, since they display a neglectable cytotoxicity and in vivo blood toxicity (Figures S15 and S16, Supporting Information). The blood half‐life of MON–FA–Mn that was used to investigate the in vivo biostability of MON–FA–Mn was determined from time‐dependent quantifications of Mn and Si, respectively. It is clearly found that the blood half‐life of MON–FA–Mn according to Si quantification is approximately equal to that obtained according to Mn quantification (Figure S17, Supporting Information), accounting for no Mn^2+^ leaching or capture by MTPs during blood circulation, which demonstrates the robust biostability of MON–FA–Mn. The excellent biostability is ascribed to the fact that the appropriate mesopores in MON can prohibit OMW MTPs from entering mesoporous channels and capturing Mn^2+^, displaying a spatial confinement effect. As well, the time‐dependent distributions of MON–FA–Mn in main organs were obtained (Figure S18, Supporting Information).

**Figure 6:**
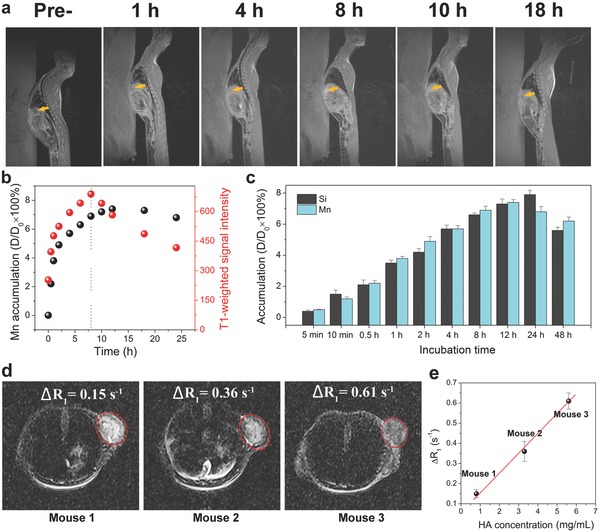
In vivo evaluation of CLRT on VX2‐bearing New Zealand rabbit. a) In vivo T1W MRI images of VX2 tumors implanted on the liver of New Zealand rabbit before and after the i.v. injection of MON–FA–Mn for different time intervals (Dose: 2 mg Mn/Kg), wherein yellow arrows indicate the VX2 tumor. b) Time‐dependent Mn accumulation in VX2 tumor and T1W signal value obtained from inset (a) c) Accumulation of Si and Mn in VX2 tumor as a function of time the i.v. injection of MON–FA–Mn for different time intervals. Data are presented as the mean ± SEM. d) In vivo T1W MRI images of VX2 tumors implanted on the subcutaneous site of nude mice before and after the i.t. injection of MON–FA–Mn and 1 h postinjection (Dose: 2 mg Mn/Kg), wherein 1/*T*_1_ in the region of interest (ROI) circled by red dotted line was determined, and ∆*R*_1_ was the difference value between postinjectio and 1 h postinjection.

T1W MRI results exhibit that the contrast and signal intensity of VX2 tumor continuously increase within 8 h, but once the intravenous (i.v.) postinjection time exceeds 8 h, the contrast and signal intensity reversely decrease, as shown in **Figure** **6**a,b. In contrast, Mn accumulation in VX2 tumors fails to decrease until the postinjection time exceeds 12 h, indicating the turning point of Mn accumulation is 4 h later than that of T1W MRI performance. This intriguing phenomenon suggests that the impairing effect toward MRI performance that is resulted from Mn^2+^ capture by HA in VX2 tumor defeats the positive effect of continuous MON–FA–Mn accumulation for intensifying MRI after 8 h. Therefore, the in vivo decreased MRI after Mn^2+^ capture by HA can realize HA detection and concurrently overcome the interference of particles' accumulation‐enhanced MRI in previous strategies. Moreover, time‐dependent accumulations of Mn and Si in tumor exhibit an inconsistent variation pace (Figure 6c), which further demonstrates Mn^2+^ detachment from MON–FA–Mn. The results sufficiently reflect the potential of CLRT for the detection of biological targets in living systems.

In general, i.v. injection disables the quantitative detection of specific targets due to unknown accumulation of probes in lesions and no constant component as the ratiometric reference,^19^ in contrast, intratumoral (i.t.) injection was universally accepted to address it.^8^ Furthermore, i.t. injection was carried out to quantitatively monitor HA and its distribution in xenografted VX2 tumor subcutaneously implanted in nude mice. It is clearly found that the T1W MRI signal is significantly intensified after i.t. injection of CLRT probes, while after 1 h postinjection, the signal drops a lot due to the presence of Mn^2+^ capture by HA (Figure S19, Supporting Information). We further change the HA levels in the mice using a different amount of hyaluronidase that can accelerate HA degradation.^20^ Comparing postinjection and 1 h postinjection of the CLRT probe, the ∆*R*_1_ values at the region of interest (ROI) in tumor are quantified to be 0.15, 0.36, and 0.61 s^−1^ (Figure 6d), and the amount of HA in each tumor is determined to be 0.8, 3.3, and 5.6 mg mL^−1^, respectively, using high‐performance liquid chromatography (HPLC).^21^ The plot of ∆*R*_1_ versus HA amount indicates that the HA level in the tumors is well represented by the T1W MRI signal (Figure 6e). The results manifest the potential of using CLRT for the quantitative analysis of various biomolecules in living systems.

## 3 Conclusion

In summary, a new MRI‐based sensing approach basing on CLRT has been established as a proof‐of‐concept to realize detection of biological targets. Two model systems, i.e., MON–FA–Mn and HMSN–NH_2_–Mn, were designed to explore the potential of CLRT, and both allow the MRI as ruler to detect two biomarkers (HA and GSH) of malignancy. Moreover, three concerns, i.e., pore diameter of mesoporous carrier and the coordination ability and spatial structure of Mn^2+^ donor (modified ligands) on CLRT probes, have been demonstrated to govern the feasibility, specificity, and generality of the CLRT sensing protocol. Therefore, rationally designing CLRT probe aiming at a certain biological target should be taken into account when using CLRT as a MRI sensing. In particular, in vitro and in vivo experiments validated the exploration of CLRT in quantitatively detecting tumor biomarkers (i.e., HA) via the decreased T1W MRI signal using the CLRT probe (i.e., MON–FA–Mn). The T1W MRI‐based CLRT can potentially provide an alternative and/or complementary approach for monitoring a variety of biological targets, because MRI can offer high‐resolution anatomical information and soft tissue contrast. However, the CLRT method is still at its infancy, and they need to be further improved for a wide window of in vivo applications involving imaging and detection of biomarkers. Also, quantitative detection represented by variations of relaxation coefficient or relaxation time needs to be further explored.

## 4 Experimental Section

*In Vitro T1W MRI of MON–FA–Mn*: The in vitro MR imaging experiment was performed on UNITED IMAGING (uMR 570, 1.5 T), and the pulse sequence used was FSE‐T1WI with the following parameters: TR = 400, TE = 20, slice thickness = 2.00 mm, matrix = 180 × 180, Acq (NEX) = 1. When using EDTA and HA as the Mn^2+^ acceptors, six groups were set, i.e., labeled number 1: MON–FA–Mn alone, labeled number 2: coexisting mixture of HA (or EDTA or GSH) or complete FBS + MON–FA–Mn, labeled number 3: HA (or EDTA or GSH) solution or complete FBS alone, labeled number 4: redispersed centrifugal precipitate after MON–FA–MN incubation with HA (or EDTA or GSH) or complete FBS, labeled number 5: centrifugal supernatant after MON–FA–MN incubation with HA (or EDTA or GSH) or complete FBS, labeled number 6: water. The concentration of MON–FA–Mn was fixed to 3 mg mL^−1^ in all corresponding groups. The concentrations of HA was 2.0 mg mL^−1^, and the concentration of GSH was 5 × 10^−3^ m. The incubation durations were 1 and 8 h when HA (EDTA or GSH) or FBS were used as the Mn^2+^ acceptors, respectively. Noticeably, Mn content in group 1 and 4 were determined by inductively coupled plasma‐atomic emission spectrometry (ICP‐AES), wherein group 1 served as the normalized standard.

Additionally, HA concentration‐dependent and Mn concentration‐dependent T1W MRI was performed on a 3.0 T clinical MRI instrument (GE Signa 3.0 T), and the pulse sequence used was a T1W FSE‐XL/90 sequence with the following parameters: TR = 1000 ms, TE = 8.1, NEX = 2. The resulting *T*_1_ values were recorded at different concentration and plotted as 1/*T*_1_ versus molar concentration of Mn, and then the slope of this line provides the longitudinal molar relaxation rate, i.e., *r*1 value. ∆*R*_1_ curve as a function of mass concentration of HA was obtained via measuring the difference of 1/*T*_1_ between pure CLRT probe and the mixture of CLRT probe and HA.

*T1W MRI and Internalization Tests in a Cellular Level*: The 10th generation VX2 cells were seeded in special 1.5 mm thickness dishes at a density of 2 × 10^5^ cells per well were cultured in 5% CO_2_ at 37 °C for 24 h, and three groups were set. The 1st group was VX2 cells after treatment with FITC‐labeled MON–FA–Mn particles (50 µg mL^−1^) for 30 min and subsequent removing unuptaken particles. The 2nd group was VX2 cells after treatment with FITC‐labeled MON–FA particles (50 µg mL^−1^) for 30 min and subsequent removing unuptaken particles. The 3rd group was VX2 cells that were first treated with excessive MnCl_2_ (100 × 10^−6^ m) for 30 min and washed with phosphate buffer solution (PBS), followed by treatment with FITC‐labeled MON–FA–Mn particles (50 µg mL^−1^) for 30 min and subsequently removing unuptaken particles. The 4th group is VX2 cells after treatment with FITC‐labeled MON–FA–Mn particles (50 µg mL^−1^) for 2 h and subsequently removing unuptaken particles. The 5th is the VX2 cells treated with the coexisting mixture of HA (2.0 mg mL^−1^) and FITC‐labeled MON–FA–Mn particles (50 µg mL^−1^, premixing duration: 30 min) for 2 h min and subsequently removing unuptaken particles. After above implementation, VX2 cells in all groups were observed on Olympus FV1000 laser scanning confocal microscope with a 40× water‐immersion objective lens.

After confocal observation, the VX2 cells in above group were digested by 0.25% trypsin (Sigma) and collected after centrifugation at a speed of 1000 rpm min^−1^. The supernatant was discarded and 1 mL PBS was added dropwise in case of VX2 cells' redispersion. These cells were taken for T1W MRI on UNITED IMAGING (uMR 570, 1.5 T).

*In Vivo T1W MRI via the I.V. Injection Manner for Qualitative HA Detection*: New Zealand rabbits bearing VX2 in situ tumor were employed as the research object. In vivo T1W MRI was performed on a 3.0 T clinical MRI instrument (GE Signa 3.0 T), and the pulse sequence used was a T1W FSE‐XL/90 sequence with the following parameters: TR = 1000 ms, TE = 8.1, NEX = 2. Scanning and image capturing of in vivo MRI were carried out before and at given time intervals after the injection of CLRT probe (i.e., MON–FA–Mn) (Mn Dose: 2 mg Mn/Kg). Animal procedures were in accordance with the guidelines of the Experimental Animal Ethics Committee of Shanghai Tenth Peoples' Hospital.

*In Vivo T1W MRI via the I.T. Injection Manner for Quantitative HA Detection*: Nude mice (BALB/c, SPF) bearing VX2 tumor was obtained via injecting 2 × 10^6^ cells into the subcutaneous site of nude mice. All experiments in nude mice were performed according to protocols approved by the Experimental Animal Ethics Committee of Shanghai Tenth Peoples' Hospital and were also in accordance with the policies of National Ministry of Health. After 2 weeks, the tumor volume reached 120 mm^3^, and then in vivo T1W MRI was carried out. Hyaluronidase with different concentrations (i.e., 0, 0.3, 1.2, 2.5 U mL^−1^, respectively) was injected into the tumor, after 8 h, CLRT probes of the same concentration (Mn Dose: 2 mg Mn/Kg) was intratumorally injected into the VX2 tumors. At three time points, i.e., preinjection, postinjection, and 1 h postinjection, T1W MRI images were acquired. The identical MRI sequences and parameters to those in aforementioned in vivo experiments were employed. HA content was detected via HPLC technology using a size exclusion column, 0.05 m phosphate buffer (pH, 5.0) mobile phase at a flow rate of 1.0 mL min^−1^ and ultraviolet absorbance detection at 200 nm.

*Statistical Analysis*: All the experiments were performed in triplicate. The obtained data were expressed as the mean value ± standard deviation (SD) and the statistical significance between two groups was analyzed by the Student's two‐tailed *t‐*test through SPS 22.0. Single, double, and triple asterisks represent *P* ≤ 0.01, 0.005, and 0.001, respectively and *p < 0.01 was considered statistically significant.

## Conflict of Interest

The authors declare no conflict of interest.

## Supporting information

Supplementary

## References

[1] a) W. R. Wilson , M. P. Hay , Nat. Rev. Cancer 2011, 11, 393; 21606941 10.1038/nrc3064

[2] J. Chen , H. Luo , Y. Liu , W. Zhang , H. Li , T. Luo , K. Zhang , Y. Zhao , J. Liu , ACS Nano 2017, 11, 12849. 29236476 10.1021/acsnano.7b08225

[3] a) K. Zhang , H. Xu , H. Chen , X. Jia , S. Zheng , X. Cai , R. Wang , J. Mou , Y. Zheng , J. Shi , Theranostics 2015, 5, 1291; 26379793 10.7150/thno.12691 PMC4568455

[4] a) H. X. Xu , M. D. Lu , L. N. Liu , Y. F. Zhang , L. H. Guo , J. M. Xu , C. Liu , Br. J. Radiol. 2012, 85, 1376; 22553290 10.1259/bjr/19932596 PMC3474036

[5] a) K. Zhang , H. Xu , X. Jia , Y. Chen , M. Ma , L. Sun , H. Chen , ACS Nano 2016, 10, 10816; 28024356 10.1021/acsnano.6b04921

[6] a) P. Mi , D. Kokuryo , H. Cabral , H. Wu , Y. Terada , T. Saga , I. Aoki , N. Nishiyama , K. Kataoka , Nat. Nanotechnol. 2016, 11, 724; 27183055 10.1038/nnano.2016.72

[7] a) V. Sayevich , C. Guhrenz , V. M. Dzhagan , M. Sin , M. Werheid , B. Cai , L. Borchardt , J. Widmer , D. R. Zahn , E. Brunner , V. Lesnyak , N. Gaponik , A. Eychmüller , ACS Nano 2017, 11, 1559; 28052188 10.1021/acsnano.6b06996

[8] J. S. Choi1 , S. Kim , D. Yoo , T. H. Shin , H. Kim , M. D. Gomes , S. H. Kim , A. Pines , J. Cheon , Nat. Mater. 2017, 16, 537. 28166216 10.1038/nmat4846

[9] a) E. J. Franzmann , G. L. Schroeder , W. J. Goodwin , D. T. Weed , P. Fisher , V. B. Lokeshwar , Int. J. Cancer 2003, 106, 438; 12845686 10.1002/ijc.11252

[10] P. Caravan , J. J. Ellison , T. J. McMurry , R. B. Lauffer , Chem. Rev. 1999, 99, 2293. 11749483 10.1021/cr980440x

[11] M. Wu , Q. Meng , Y. Chen , Y. Du , L. Zhang , Y. Li , L. Zhang , J. Shi , Adv. Mater. 2015, 27, 215. 25423915 10.1002/adma.201404256

[12] a) T. D. Macdonald , T. W. Liu , G. Zheng , Angew. Chem., Int. Ed. 2014, 53, 6956; 10.1002/anie.201400133 24840234

[13] a) C. Platas‐Iglesias , D. Esteban‐Gomez , L. Helm , M. Regueiro‐Figueroa , J. Phys. Chem. A﻿﻿ 2016, 120, 6467; 27459626 10.1021/acs.jpca.6b05423

[14] R. Kripal , A. K. Shukla , Spectrochim. Acta, Part A 2007, 66, 453. 10.1016/j.saa.2006.03.022 17023197

[15] C. Huang , Z. Tang , Y. Zhou , X. Zhou , Y. Jin , D. Li , Y. Yang , S. Zhou , Int. J. Pharm. 2012, 429, 113. 22406331 10.1016/j.ijpharm.2012.03.001

[16] Y. J. Kim , R. C. Johnson , J. T. Hupp , Nano Lett. 2001, 1, 165.

[17] a) K. Zhang , H. Chen , F. Li , Q. Wang , S. Zheng , H. Xu , M. Ma , X. Jia , Y. Chen , J. Mou , X. Wang , J. Shi , Biomaterials 2014, 35, 5875; 24746229 10.1016/j.biomaterials.2014.03.043

[18] K. Zhang , H. Chen , X. Zhou , Y. Gong , G. Zhang , X. Wang , Y. Chen , J. Shi , J. Mater. Chem. A﻿﻿ 2014, 2, 1515.

[19] Y. Hou , J. Zhou , Z. Gao , X. Sun , C. Liu , D. Shangguan , W. Yang , M. Gao , ACS Nano 2015, 9, 3199. 25670342 10.1021/acsnano.5b00276

[20] D. C. West , I. N. Hampson , F. Arnold , S. Kumar , Science 1985, 228, 1324. 2408340 10.1126/science.2408340

[21] J. Roboz , J. Greaves , D. Silides , A. P. Chahinian , J. F. Holland , F. James , Cancer Res. 1985, 45, 1850. 3978644

