# Supplementary material for: Coordination‐Responsive Longitudinal Relaxation Tuning as a Versatile MRI Sensing Protocol for Malignancy Targets
Source: Adv Sci (Weinh). 2018 Jul 10;5(9):1800021. doi: 10.1002/advs.201800021 (PMC6145269; doi:10.1002/advs.201800021)
Supplement: Supplementary file 1 — Supplementary [file ADVS-5-1800021-s001.pdf]

Copyright WILEY-VCH Verlag GmbH & Co. KGaA, 69469 Weinheim, Germany, 2018.

ADVANCED SCIENCE
Open Access

Supporting Information

for *Adv. Sci.,* DOI: 10.1002/advs.201800021

Coordination-Responsive Longitudinal Relaxation Tuning as a Versatile MRI Sensing Protocol for Malignancy Targets

Kun Zhang, Yu Cheng, Weiwei Ren, Liping Sun, Chang Liu, Dan Wang, Lehang Guo, Huixiong Xu,* and Yongxiang Zhao**

Copyright WILEY-VCH Verlag GmbH & Co. KGaA, 69469 Weinheim, Germany, 2016.

Supporting Information

Coordination-responsive Longitudinal Relaxation Tuning as a Versatile MRI Sensing Protocol for Malignancy Targets

Kun Zhang, Yu Cheng, Weiwei Ren, Liping Sun, Chang Liu, Dan Wang, Lehang Guo, and Huixiong Xu**

Part A: materials and methods

Materials. Tetraethyl orthosilicate (TEOS), triethanolamine (TEA), ethanol and hydrochloric acid (HCl, 37%) were purchased from Sinopharm Chemical Reagent Co.. Bis[3-(triethoxysilyl)propyl]tetrasulfide (BTES), cetyltrimethylammonium chloride (CTAC, 25 wt%), 3-aminopropyltriethoxysilane (APTES), fluorescein isothiocyanate (FITC), 1-ethyl-3-(3-dimethly-aminopropyl) carbodiimide (EDC), N-hydroxysuccinimide (NHS), folic acid (FA), ethylenediaminetetraacetic acid (EDTA), hyaluronic acid sodium salt (MW=8000~15000), $MnCl_2$ (AR) and $FeCl_3·6H_2O$ (AR) were obtained from Sigma-Aldrich Co., Ltd.. Phosphate buffer solution (PBS, pH=7.4) was obtained from Shanghai Runcheng Biomedical Co., Ltd.. Deionized water was used in all experiments. Fetal bovine serum (FBS) was obtained from Sijiqing Biological Engineering Materials Co., Ltd., Hangzhou. All chemicals were used as received without further purification.

Characterizations. Transmission electron microscopy (TEM) images were acquired on a JEM-2100F electron microscope operated at 200 kV. Scanning electron microscopy (SEM) images/scanning transimission electron microscopy (STEM) images and corresponding element mapping/EDS spectrum were obtained on a field emission Maggellan 400 microscope (FEI Company). Nitrogen adsorption-desorption isotherms and pore size distribution at 77 K

were tested on a Micromeritics Tristar 3000 analyzer. All samples were pre-treated for 4 h at 423 K under nitrogen conditions for removing adsorbed H_2O and O_2. The pore size distribution was calculated according to the desorption branch of MON isotherms by the Barrett-Joyner-Halenda (BJH) method. Specific surface areas were calculated by the Brunauer Emmet Teller (BET) method. Ultraviolet/Visible (UV/Vis) spectra were recorded on a UV-3101PC Schimadzu spectroscope. Dynamic light scattering (DLS) measurement was used to determine the hydrodynamic particle sizes and zeta potentials of MON, MON-NH_2 and MON-FA on Zetasizer Nanoseries (Nano ZS90). Inductively coupled plasma atomic emission spectrometry (ICP-AES) test was conducted on Agilent 725 (Agilent company, American) for quantifying Si and Mn. The CLSM images for evaluating FA targeting were obtained in FV 1000, Olympus. TG analysis was carried out to determine the loading amount of FA on TG-DSC analyzer (STA449C, NETZSCH-Gerätebau GmbH). X-ray photoelectron spectroscopy (XPS) was acquired to determine the valence state of Mn^{2+} on an ESCALAB 250Xi X-ray photoelectron spectroscopy. Electron spin resonance (ESR) spectra of detecting Mn^{2+} were recorded on on a Bruker EMX-8/2.7 spectrometer. Fourier transform infrared (FTIR) spectra was measured on a Nicolet Avatar 370 FT-IR spectrophotometer using KBr pellet as reference to demonstrate the modifications of MON by -NH_2 and HA. Raman spectra were recorded on a DXR Microscope (Thermo Scientific) with an excitation length of 532 nm.

Synthesis of MONs and FITC-labeled MONs

MONs can be obtained according to a well-established method.[1] In detail, cetyltrimethylammonium chloride (CTAC, 2 g) and triethanolamine (TEA) were dissolved in 20 mL of water at 95 °C. After 20 min stirring, an aqueous mixture consisting of 1 g tetraethylorthosilicate (TEOS) and 1.3 g bis[3-(triethoxysilyl)propyl]tetrasulfide (BTES) was added at a rate of 20 mL/min using an injection pump and the reaction was carried out for

another 4 h under magnetic stirring. The resultants were harvested *via* the centrifugation method and rinsed for several times with ethanol and water in turns to remove the unreacted precursors. Subsequently, the collected MONs were extracted three times for an overnight per time using hydrochloric acid (HCl) solution in ethanol (10% v/v) at 75 °C by refluxing to remove CTAC.

In addition, fluorescein isothiocyanate (FITC)-labeled MONs (FITC-MONs) were obtained by the co-condensation of FITC-labeled 3-aminopropyltriethoxysilane (APTES, FITC-APTES), TEOS and BTES, and other parameters conformed to aforementioned procedures. Thereinto, FITC-APTES was yielded by the reaction between fluorescein isothiocyanate (FITC) isomer (20 mg) and excessive APTES (200 μL) in ethanol under light-sealed and dry-conditions for 12 h at room temperature.

Synthesis of amino-functionalized MONs (MONs-NH_2)

Firstly, the surface of MONs was functionalized with amine groups by treatment with APTES. 50 mg of MONs were first dispersed in 100 mL of ethanol, and then the solution was refluxed for 12 h (78 °C), followed by the addition of 50 μL of APTES. After centrifugation and rinsing with water and ethanol, amine-functionalized MONs (MONs-NH_2) were re-dispersed in water for next HA chelation.

Modification of MON-NH_2 by FA (MON-FA)

A certain amount of MON-NH_2 was dispersed in 50 mL 2-(N-morpholino)ethanesulfonic acid (MES, 0.1 M, pH=6) buffer under stirring at room temperature for another 2 h after ultrasonication dispersion for 10 min, followed by addition of excessive NHS (100 mg) and EDC for activating -NH_2. After 30 min, excessive FA (100 mg) was added and reacted for 12 h at room temperature under continuously stirring. Afterwards, the HA-modified MON (MON-FA) were collected *via* the high-speed centrifugation method, during which these

particles were rinsed 3 times with water to remove unreacted FA, EDC and NHS. Ultimately, the obtained MON-FA was dried *via* freeze-drying method under vacuum condition.

Chelation of Mn^{2+} on MON-FA (MON-FA-Mn^{2+})

Aforementioned MON-FA (20 mg) were re-dispersed in deionized water (50 mL), and then 200 mg of $MnCl_2$ was added and stirred for 12 h so as to sufficiently coordinate with MON-FA, yielding MON-FA-Mn. Subsequently, MON-FA-Mn was harvested *via* the centrifugation method and rinsed three times with deinoized water to remove uncoordinated Mn^{2+}.

Synthesis of HMSN, HMSN-NH_2 and HMSN-NH_2-Mn.

HMSNs with 400 nm in diameter and a mesopores of less than 3 nm were obtained according to a previous report.[2] In detail, a mixture solution of 35.7 mL ethanol, 5 mL deionized water, and 1.57 mL ammonia solution were gently stirred and incubated at 30 °C for 0.5 h. After that, 3 mL of pre-warmed TEOS at 30 ºC was poured quickly into above mixture and continuously stirred for 1 h. Subsequently, milky colloidal silica nanospheres (s-SiO_2) were collected and washed with EtOH and deionized water for twice and once, respectively. The nanospheres were dispersed into 14 mL of deionized water by ultrasonication, of which 2 mL dispersion was taken out and diluted with 8 mL deionized water. Next, the 10 mL dispersion was dropwise added into the another mixture consisting of 15 mL EtOH, 15 mL deionized water, 75 mg CTAB and 0.275 mL ammonia solution, and stirred for 30 min. After that, 0.130 mL of TEOS was dropwise added into this mixture, and stirred for another 3 h, giving rise to the core/shell structured silica nanoparticles. Afterwards, these particles were collected by high-speed centrifugation, and then were re-dispersed in 20 ml Na_2CO_3 aqueous solution (0.4 M) for 15 h at 60 °C. Further, these obtained HMSN were rinsed for at least three times using deionized water and ethanol. Ultimately, the CTAB templates in as-obtained HMSNs were removed by extraction with EtOH containing 10% HCl for 24 h per time and 3 times in sum at 68 °C. The CTAB-extracted HMSNs were dried *via* the freeze-drying method under vacuum.

The synthesis procedure of HMSN-NH_2 was approximately identical to that of MON-NH_2. As for HMSN-NH_2-Mn, the synthesis process was also identical to that of MON-FA-Mn, and the difference lies in that MON-FA was replaced by HMSN-NH_2.

Release measurement of Mn^{2+} from MON-FA-Mn in fetal bovine serum (FBS).

10 mg MON-FA-Mn was uniformly dispersed in 2 mL FBS, and then added in the dialysis bag (cutoff molecule: 3000) that was sealed by a clip, and the whole dialysis bag was placed in an opaque centrifugal tube full of 23 mL FBS. Ultimately, the centrifugal tube was fastened in an electronic shaking table with an oscillation rate of 150 rpm/min at 37 °C. At several certain intervals, 1 mL releasing solution was taken out and diluted into 6 mL for ICP-AES measurement for determining Mn content and Mn release profile, during which 1 mL of fresh FBS was supplemented into the tube.

***In vitro* T1W MRI of HMSN-NH_2-Mn.**

The apparatus parameters using HMSN-NH_2-Mn were identical to those of *in vitro* MRI using MON-FA-Mn. Similarity, 6 groups were set, *i.e.*, labeled number 1: HMSN-NH_2-Mn alone, labeled number 2: co-existing mixture of GSH (or HA) + HMSN-NH_2-Mn, labeled number 3: pure GSH (or HA) solution, labeled number 4: re-dispersed centrifugal precipitate after HMSN-NH_2-Mn incubation with GSH (or HA), labeled number 5: centrifugal supernatant after HMSN-NH_2-Mn incubation with GSH (or HA), labeled number 6: water. The concentration of HMSN-NH_2-Mn was fixed to 3 mg/mL in all corresponding groups, and the incubation duration was 1 h. In particular, the concentrations of GSH and HA was 0.5 mg/mL and 5 mM, respectively. Noticeably, Fe content in group 1 and 4 were determined by ICP-AES, wherein group 1 served as the normalized standard.

Blood biocompatibility assessment of MON-FA-Mn *in vivo.*

New Zealand rabbit weighing 2.0 Kg in average were provided by the Laboratory Animal Center of Shanghai Tenth Peoples' Hospital (Shanghai, China). All animal procedures were performed under the guideline approved by the Experimental Animal Ethics Committee of

Shanghai Tenth Peoples' Hospital, Tongji University School of Medicine, and accept their supervision and inspection. MON-FA-Mn (dose: 2 mg Mn/Kg) were intravenously injected into rabbit, and this group served as the experimental group. The rabbit with injected PBS were selected as the control. Blood samples were harvested on 30 and 60 days after post-injection.

Cell culture.

New Zealand rabbit with bearing VX2 in leg muscle were supplied by Laboratory Animals Center of Tenth Peoples' Hospital of Tongji University. The peripheral fresh VX2 tissues were collected and washed 3 times with PBS, and then chopped into some small tissues (0.5 mm - 1.00 mm). The small tissues were digested using Tyrisin (2.5 mg/mL) and collagenase (1 mg/mL) for 20 min at 37 °C. The digested tissues were filtrated by 200-mesh nylon sieve to remove large agglomerates of VX2 cells, and the filtered cells were collected *via* centrifugation at a speed of 1000 rpm/min for 50 min. The first-generation VX2 cells were then cultured in the RPMI 1640 medium containing 10% FBS and 1% antibiotics (streptomycin and penicillin) (Invitrogen). After the cells were grown to the 10th generation, cell experiment and in-situ liver VX2 model establishment were carried out. All cells were maintained at 37 °C in a humidified atmosphere of 5% CO_2 incubator, and cells were harvested using 0.25% trypsin (Sigma).

***In vivo* bio-distributions of MON-FA-Mn in mains organs and VX2 tumors of New Zealand rabbit.**

All *in vivo* animal experiments in New Zealand rabbits were performed according to protocols approved by the Experimental Animal Ethics Committee of Shanghai Tenth Peoples' Hospital and were in accordance with the policies of National Ministry of Health. New Zealand rabbits bearing VX2 in-situ tumor were randomly divided into several groups (n=3) according to the set time intervals (5 min, 10 min, 0.5 h, 1 h, 2 h, 4 h, 8 h, 12 h, 24 h, 48 h). MON-FA-Mn dispersion in PBS (Mn Dose: 1 mg Mn/Kg) was injected into New Zealand rabbit *via* the back

ear edge vein. Under the fixed time intervals, the rabbits were euthanized *via* injecting excessive anesthetics (2.5% pentobarbital), and VX 2 tumor and other organs (heart, liver, spleen, lung and kidney) of each rabbit were isolated and digested by chloroazotic acid. Subsequently, Mn and Si were measured *via* the ICP-AES method, and the results can be transformed into the accumulation percentage according to the initial injection dose.

Blood half-life of MON-FA-Mn using Si and Mn as the detection references. Healthy New Zealand rabbit weighing 2.0 Kg in average were used. 4 mL of MON-FA-Mn dispersion (7.4 mg/mL) was injected into healthy New Zealand rabbit *via* the back ear edge vein. At each certain time interval, 1 mL of blood were extracted from the arteria femoralis of rabbits *via* the vacuum blood collection tube, and then digested by the chloroazotic acid. Subsequently, the time-dependent variation profiles of Mn and Si in blood were measured *via* the ICP-AES method, and the blood half-life can be obtained *via* the fitting curve.

Animal model establishment.

New Zealand rabbit with an average body weight of about 2 Kg were supplied by Laboratory Animals Center of Tenth Peoples' Hospital of Tongji University, and were kept in sterilized cages with supply of filtered air, sterile food, and water. Before any experiment, the rabbits experienced the anesthesia treatment *via* injecting 2.5 mL pentobarbital (2.5%). The 10th generation VX2 cells were re-suspended in PBS with a density of 1×10^{6}/mL after digestion by 0.25% trypsin (Sigma). Firstly, rabbit model with leg muscle-bearing VX2 tumor was established, and 4 mL of cell suspension was injected into the front leg muscle, and the tumor growth was monitored. When the tumor was grown into above 1 cm in diameter, the tumor was taken out and chopped into some smaller tissue blocks (< 1 mm). The abdomen of anaesthetic rabbits were split with a small cut, and then the chopped tumor tissue were embedded into the deep position of liver *via* the biopsy needle. Ultimately, the wound was sutured. The whole in-situ VX2 tumor establishment was implemented in an asepsis environment. About 2 weeks later, the rabbit was monitored by MRI technology to determine

whether the in-situ VX2 tumor was successful. When the tumor grew to 0.6 mm, the *in vivo* MRI experiment was carried out. All *in vivo* animal experiments were performed according to protocols approved by the Experimental Animal Ethics Committee of Shanghai Tenth Peoples' Hospital and were in accordance with the policies of National Ministry of Health.

References

[1] M. Wu, Q. Meng, Y. Chen, Y. Du, L. Zhang, Y. Li, L. Zhang, J. Shi, *Adv. Mater.* **2015**, *27*, 215.

[2] K. Zhang, H. Xu, H. Chen, X. Jia, S. Zheng, X. Cai, R. Wang, J. Mou, Y. Zheng, J. Shi, *Theranostics* **2015**, *5*, 1291;

Part B: Supplementary figures

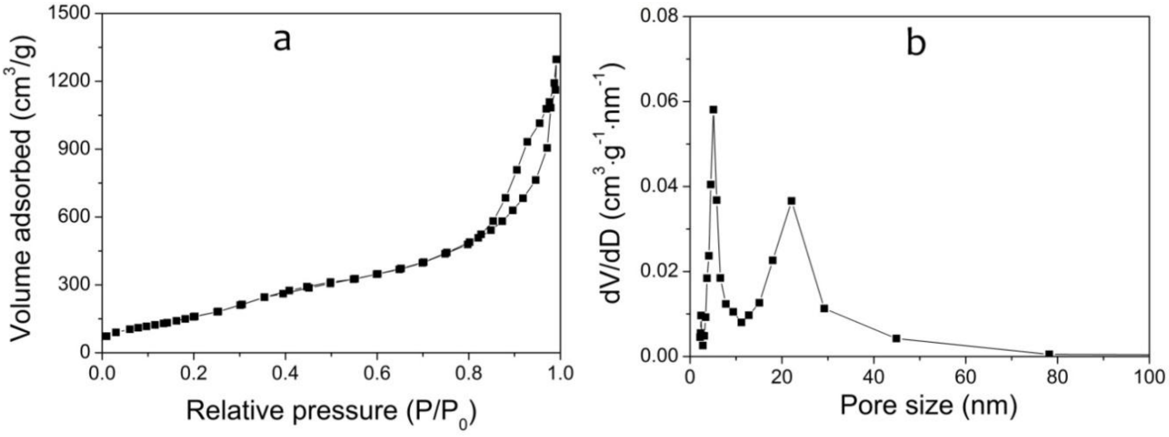

Figure S1 N_2 adsorption-desorption isotherm (a) and pore size distribution (b) of MON-FA-Mn.

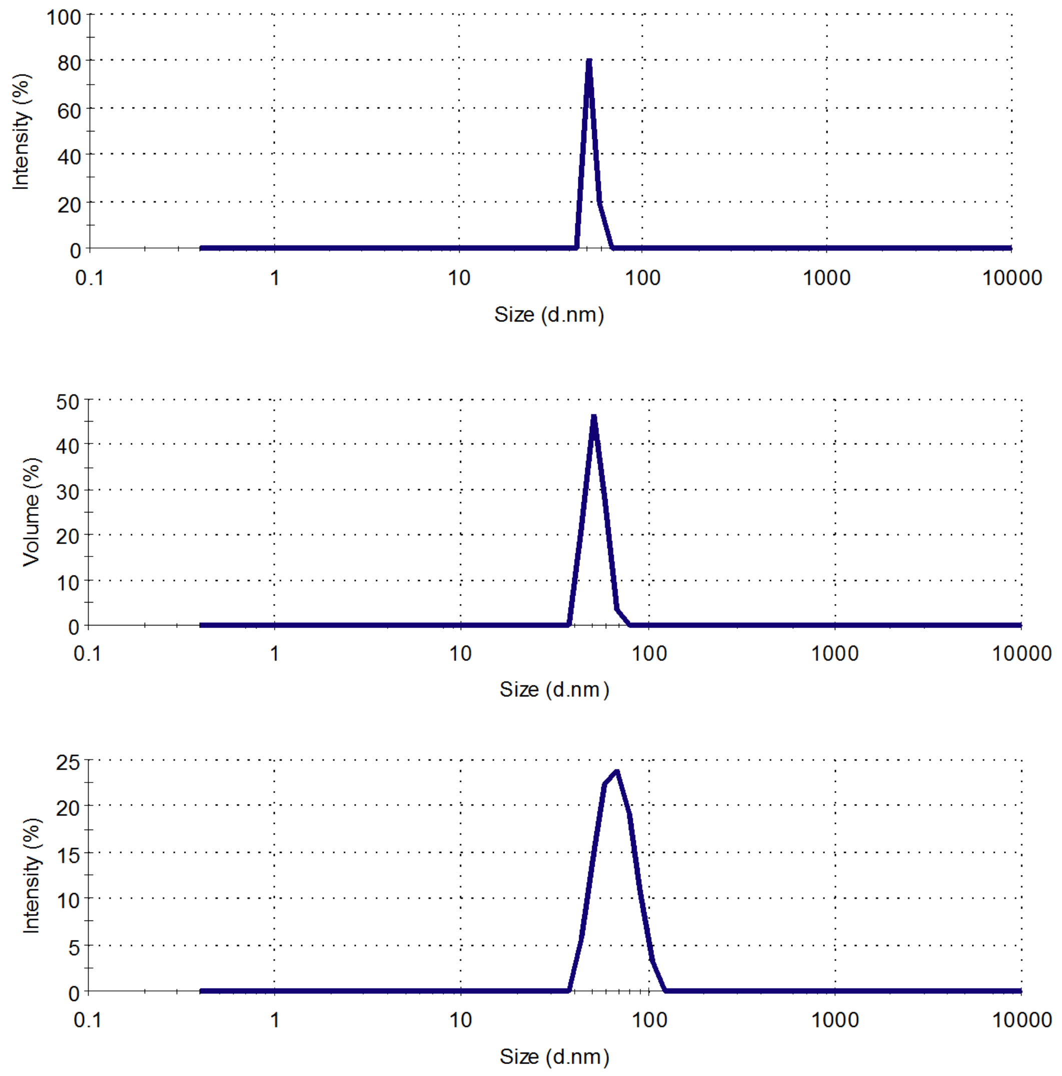

Figure S2 Size distributions of MON carrier, MON-NH_2 and MON-FA, which were obtained *via* the dynamic light scattering (DLS) method.

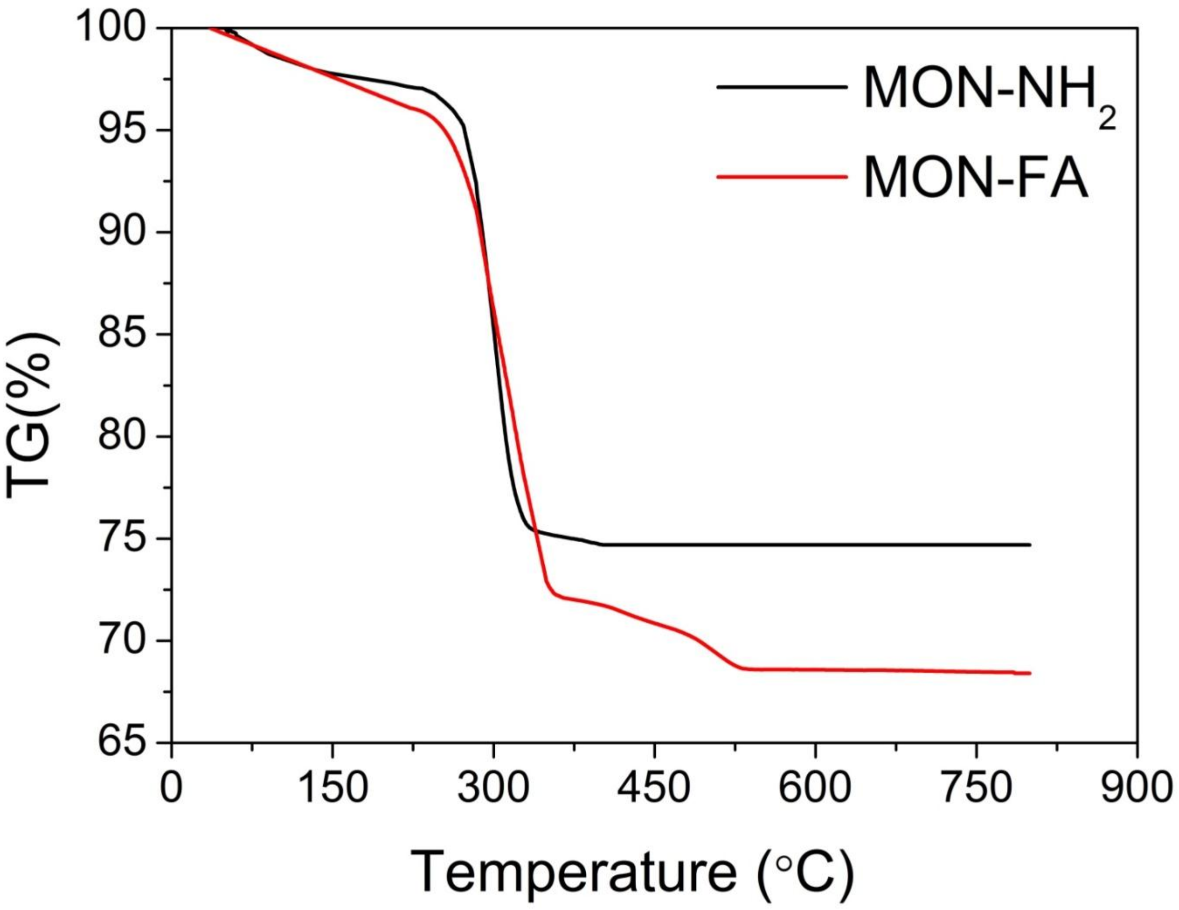

Figure S3 Thermogravimetric analysis of MON-NH_2 and MON-FA.

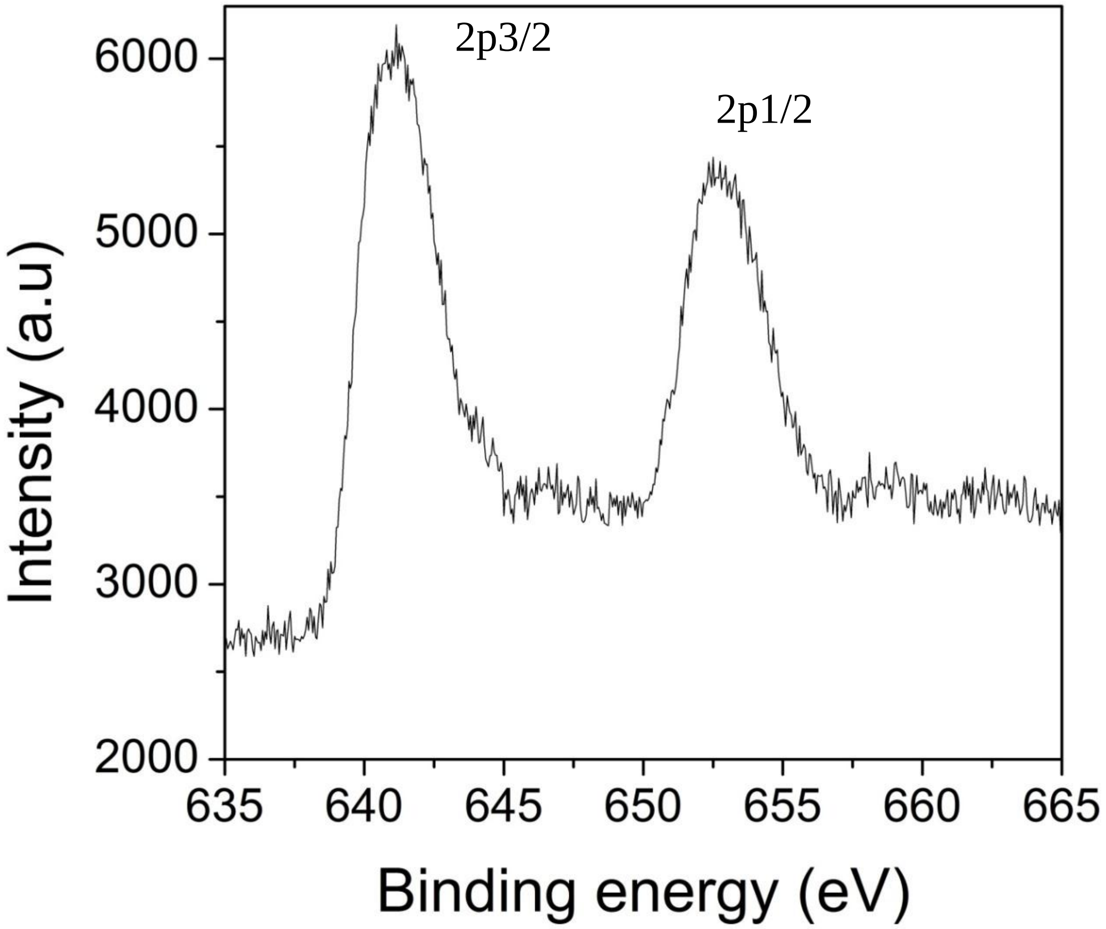

Figure S4 XPS spectrum of MON-FA-Mn, and the characteristic peak of Mn^{2+} is observed at 641.1 eV of 2p1/2 spectral line and 653.4 eV of 2p1/2 spectral line.

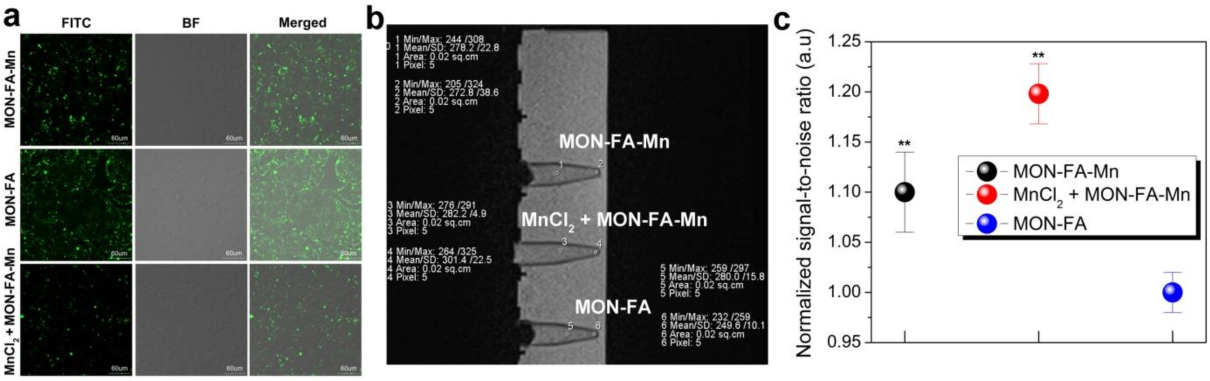

Figure S5 (a) Confocal images of VX2 cells after three different treatments, *i.e.*, MON-FA, MON-FA-Mn and $MnCl_2$ + MON-FA-Mn; (b) T1W MRI of collected cells obtained from (a); (c) Corresponding normalized signal-to-noise ratio of VX2 cells (2, 4, 6) to the added PBS medium (1, 3, 5) obtained from b. Data are presented as the mean ± SEM.

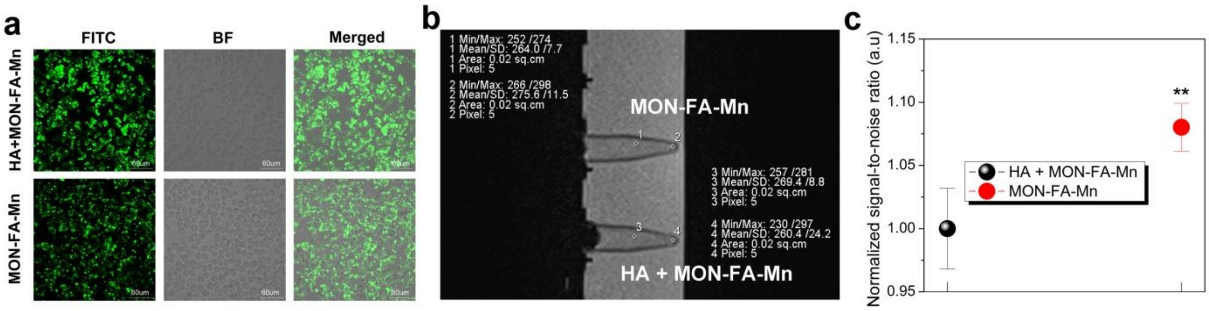

Figure S6 (a) Confocal images of VX2 cells after two different treatments, *i.e.*, MON-FA-Mn and HA + MON-FA-Mn; (b) T1W MRI of collected cells obtained from (a); (c) Corresponding normalized signal-to-noise ratio of VX2 cells (2, 4) to the added PBS medium (1, 3) obtained from b. Note '**' represents $P< 0.005$. Data are presented as the mean ± SEM.

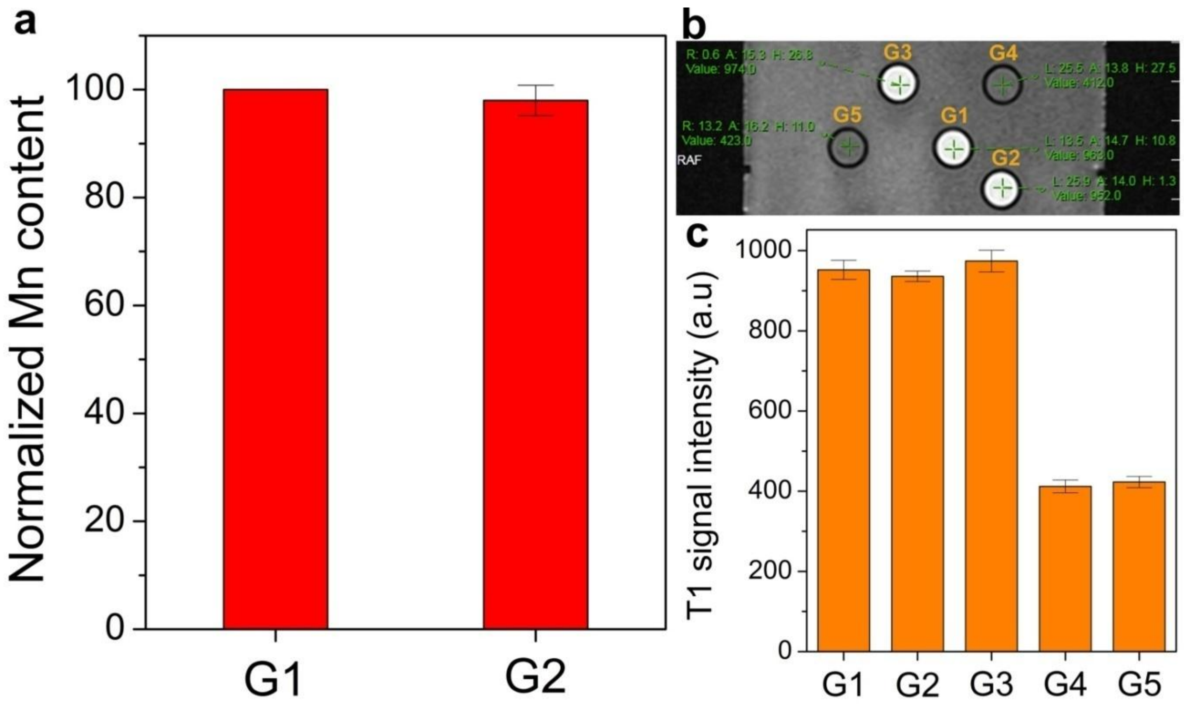

Figure S7 (a) Normalized Mn content of G1 and G2 *via* the ICP-AES method (b) T1-weighted MRI images of different groups (G1-G5), (b) the corresponding T1-weighted signal intensity of G1-G5. G1-G5 represent the initial MON-FA-Mn, the mixture of MON-FA-Mn and GSH, the re-dispersed centrifugal precipitate after MON-FA-Mn incubation with HA, the centrifugal supernatant after MON-FA-Mn MON-FA-Mn incubation with HA, and HA solution, respectively.

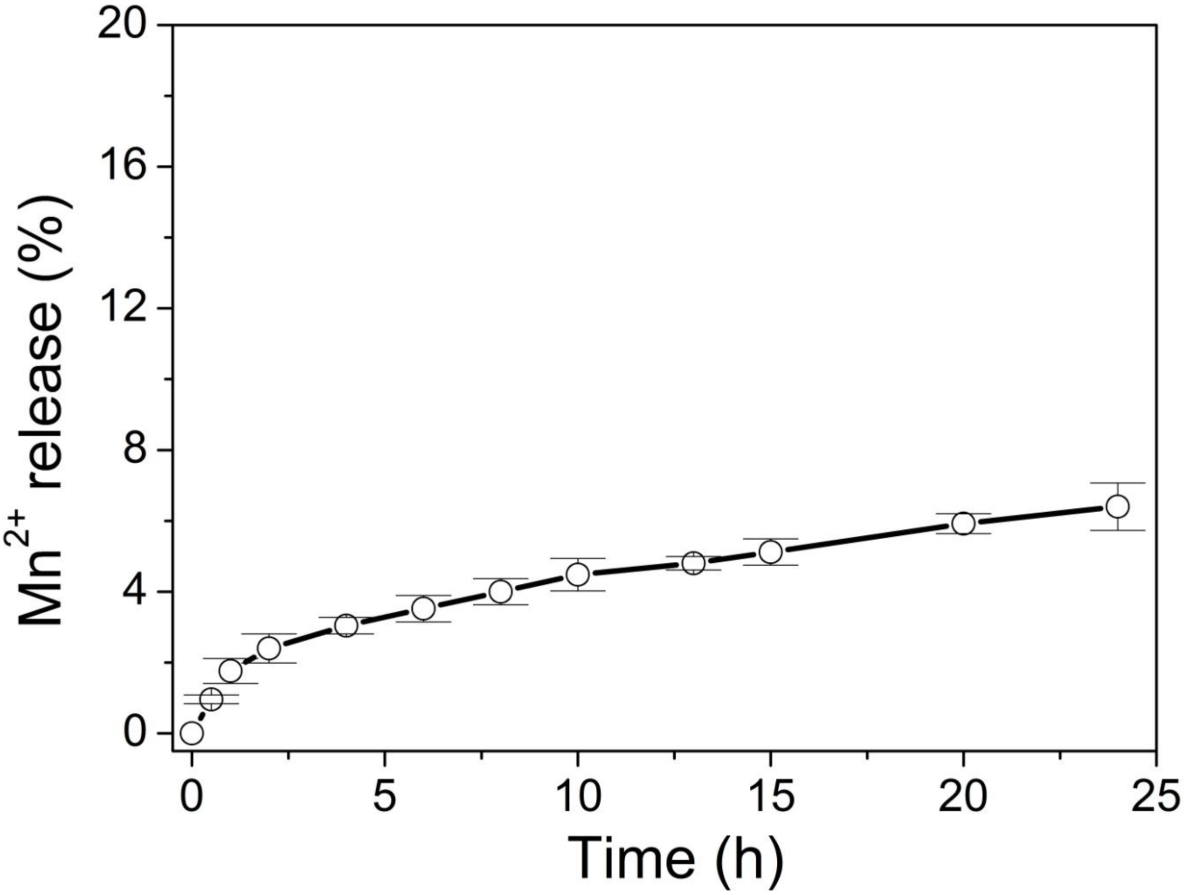

Figure S8 Mn release profile from MON-FA-Mn when FBS served as the dispersion medium.

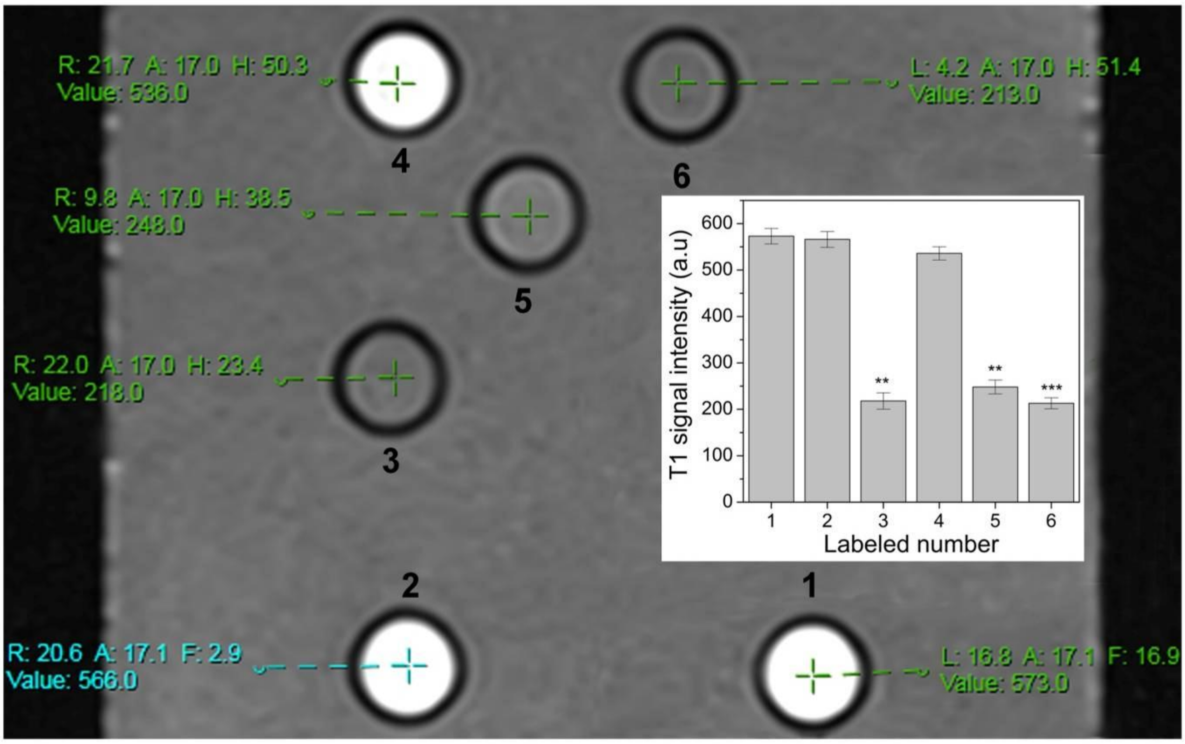

Figure S9 T1-weighted MRI image of different labeled groups 1-6, and the inset is the quantification T1 signal intensity of these labeled groups. Labeled group 1-6 represent the initial MON-FA-Mn, the mixture of MON-FA-Mn and FBS (5 μM), FBS alone, the re-dispersed centrifugal precipitate after MON-FA-Mn incubation with FBS, the centrifugal supernatant after MON-FA-Mn incubation with FBS, and the control (water), respectively. Note: '**' and '***' represent $P<0.005$ and 0.001, respectively.

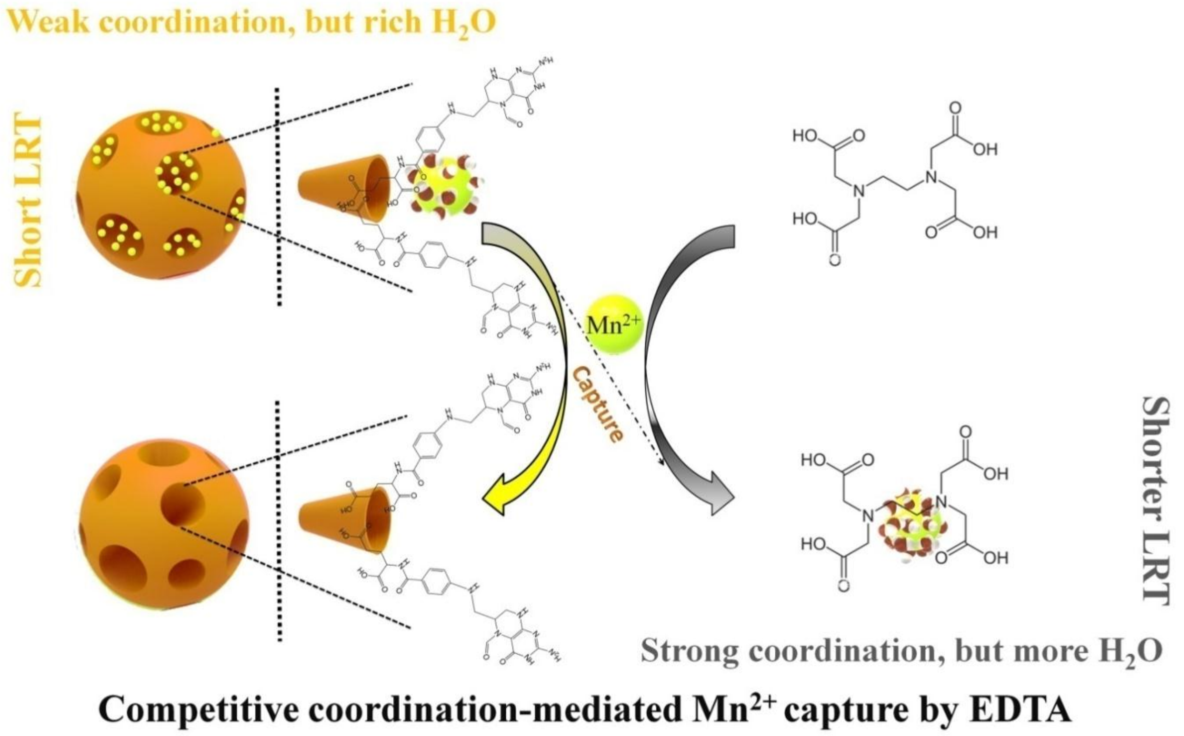

Figure S10 Operation schematic of EDTA monitoring through the CLRT-based T1W MRI sensing protocol using MON-FA-Mn as probe, in which LRT represents longitudinal relaxation time.

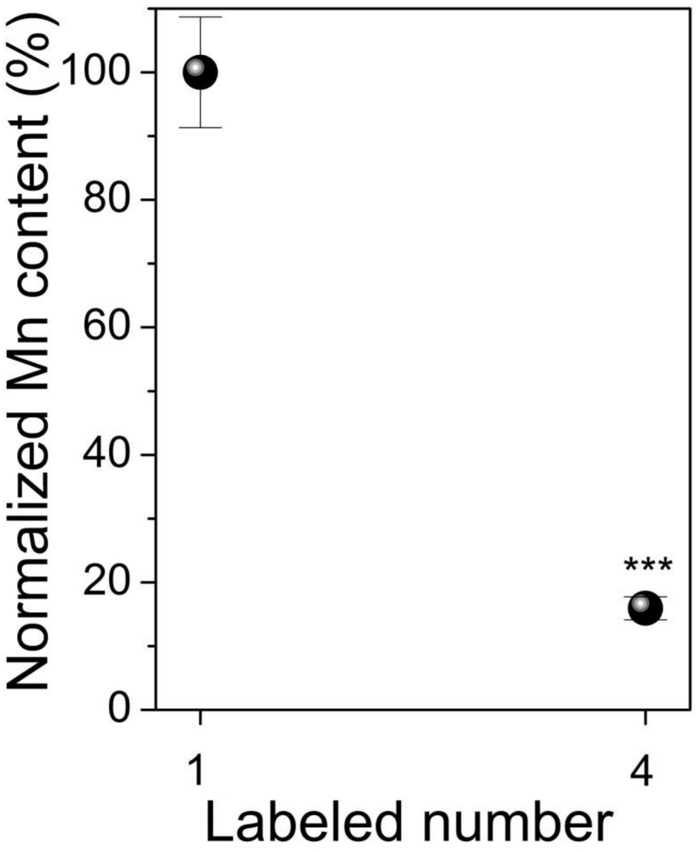

Figure S11 Normalized Mn content of labeled number 1 and 4 *via* the ICP-AES method, wherein labeled number 1 represents the initial MON-FA-Mn, and labeled number 4 represent the re-dispersed centrifugal precipitate after MON-FA-Mn incubation with EDTA. Note: '***' represents $P < 0.001$.

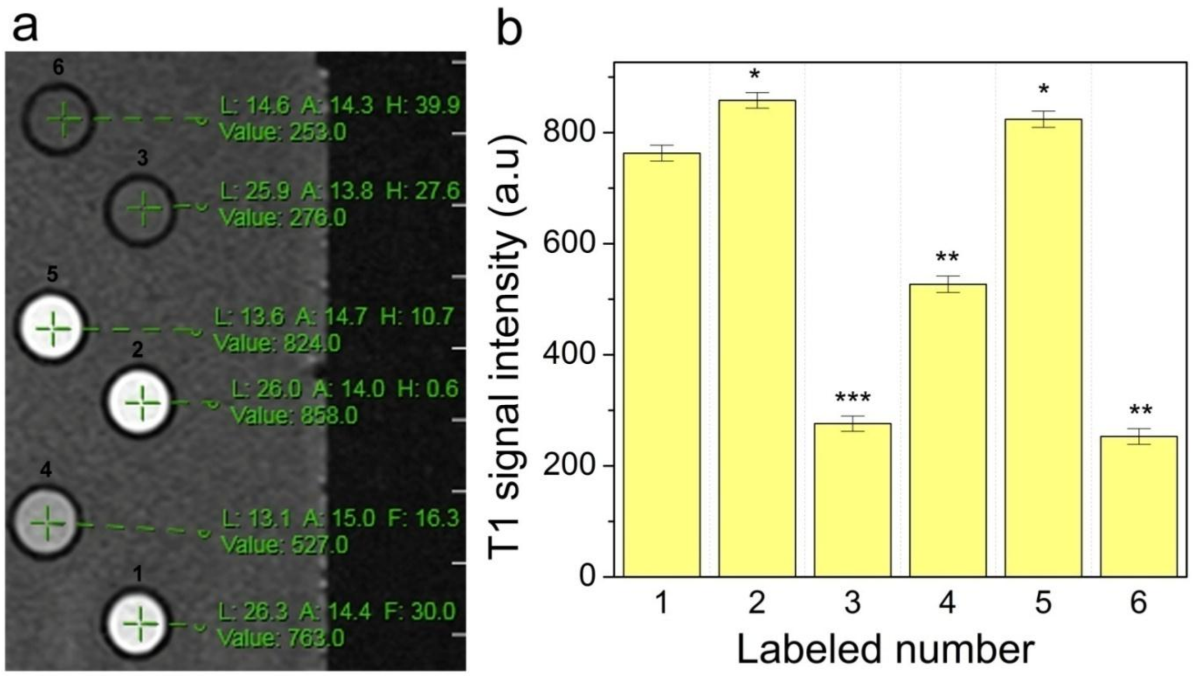

Figure S12 (a,b) T1-weighted MRI images (a) and corresponding T1-weighted signal intensities (b) of labeled number 1 - 6, wherein labeled number 1-6 represent the initial MON-FA-Mn, the mixture of MON-FA-Mn and EDTA, EDTA solution, the re-dispersed centrifugal precipitate after MON-FA-Mn incubation with EDTA the centrifugal supernatant after MON-FA-Mn incubation with EDTA, and the control (water), respectively**.** Note: '*', '**' and '***' represent P<0.01, 0.005 and 0.001, respectively.

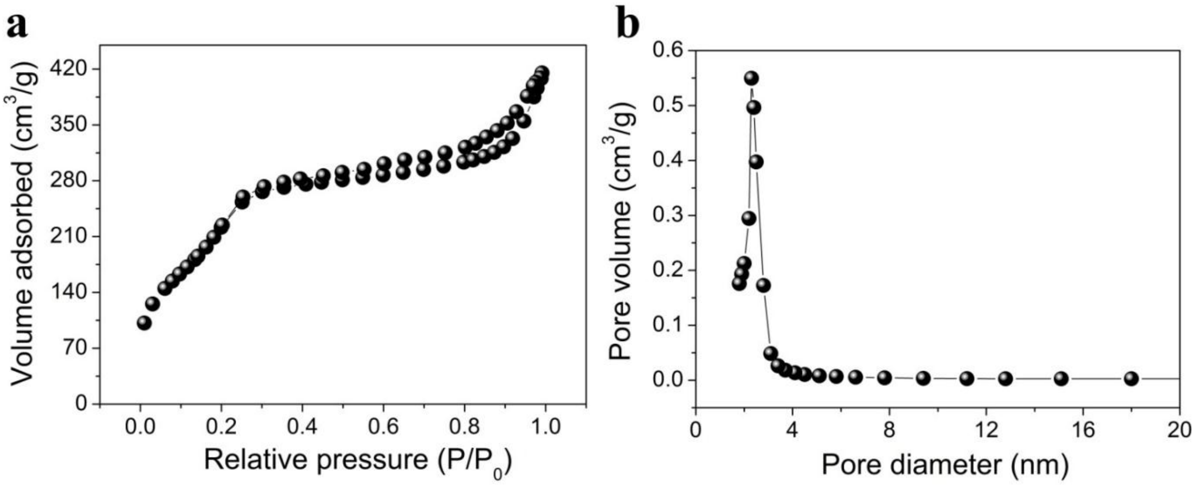

Figure S13 N_2 adsorption-desorption isotherm (a) and pore size distribution (b) of HMSN-NH_2-Mn.

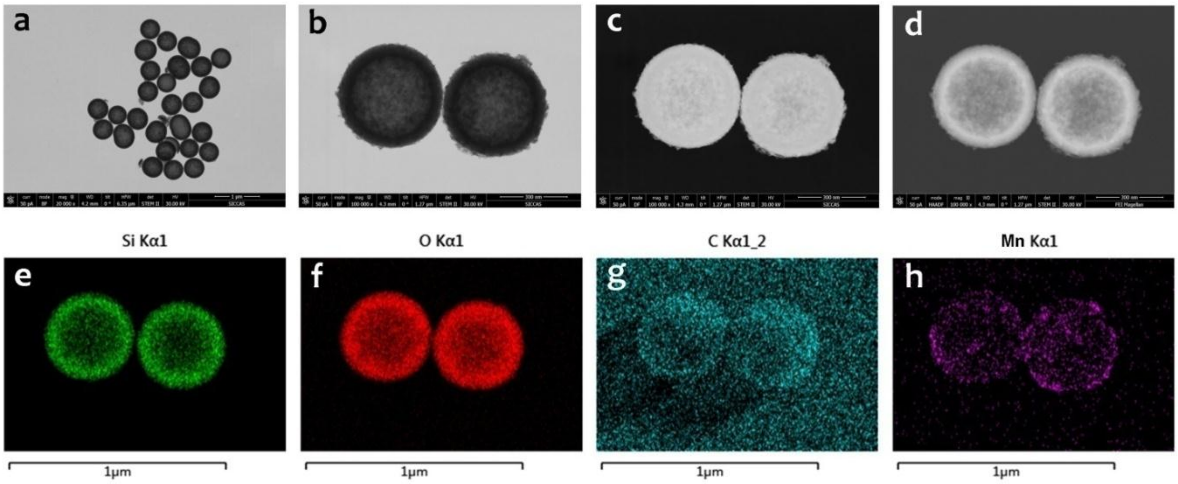

Figure S14 (a-d) TEM (a,b), bright-field (BF, c) and dark-field (DF, d) images of HMSN-NH_2-Mn, (e-h) Atom map scanning of HMSN-NH_2-Mn, and image e-h represent Si, O, C and Mn, respectively.

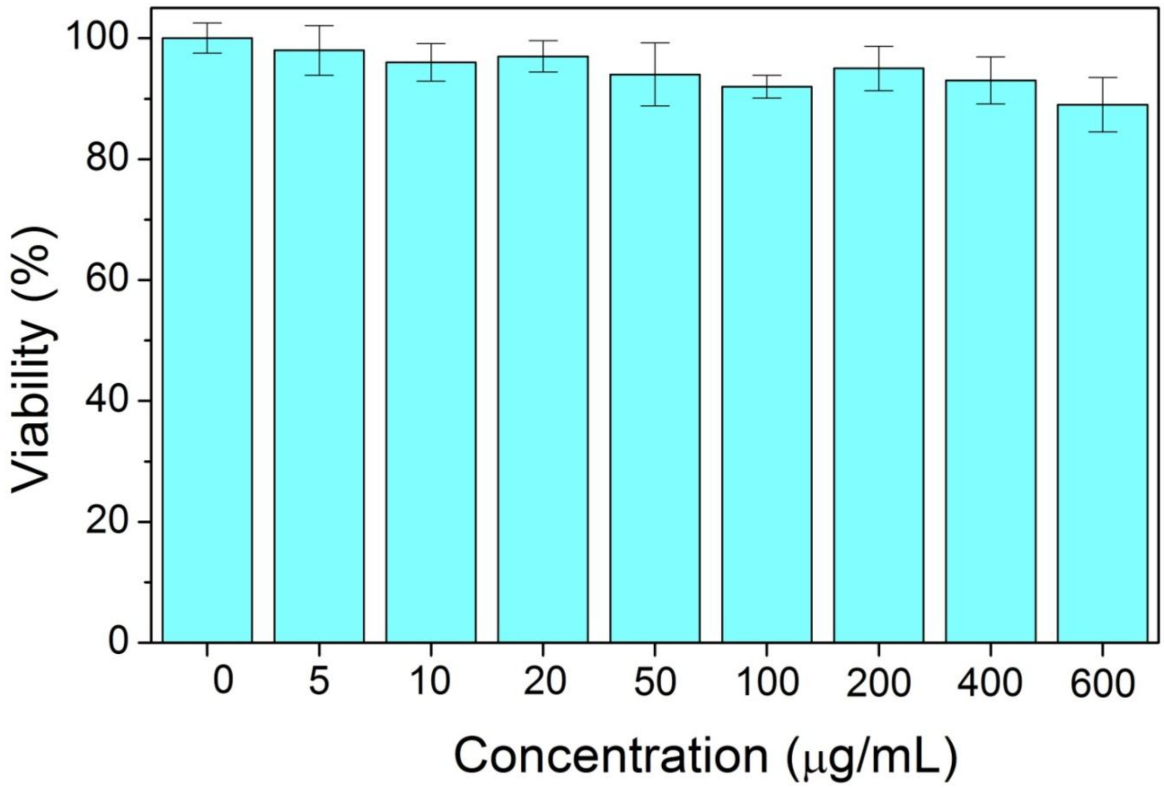

Figure S15 Cell viability of L929 cells after incubation with MON-FA-Mn at varied mass concentration for 48 h.

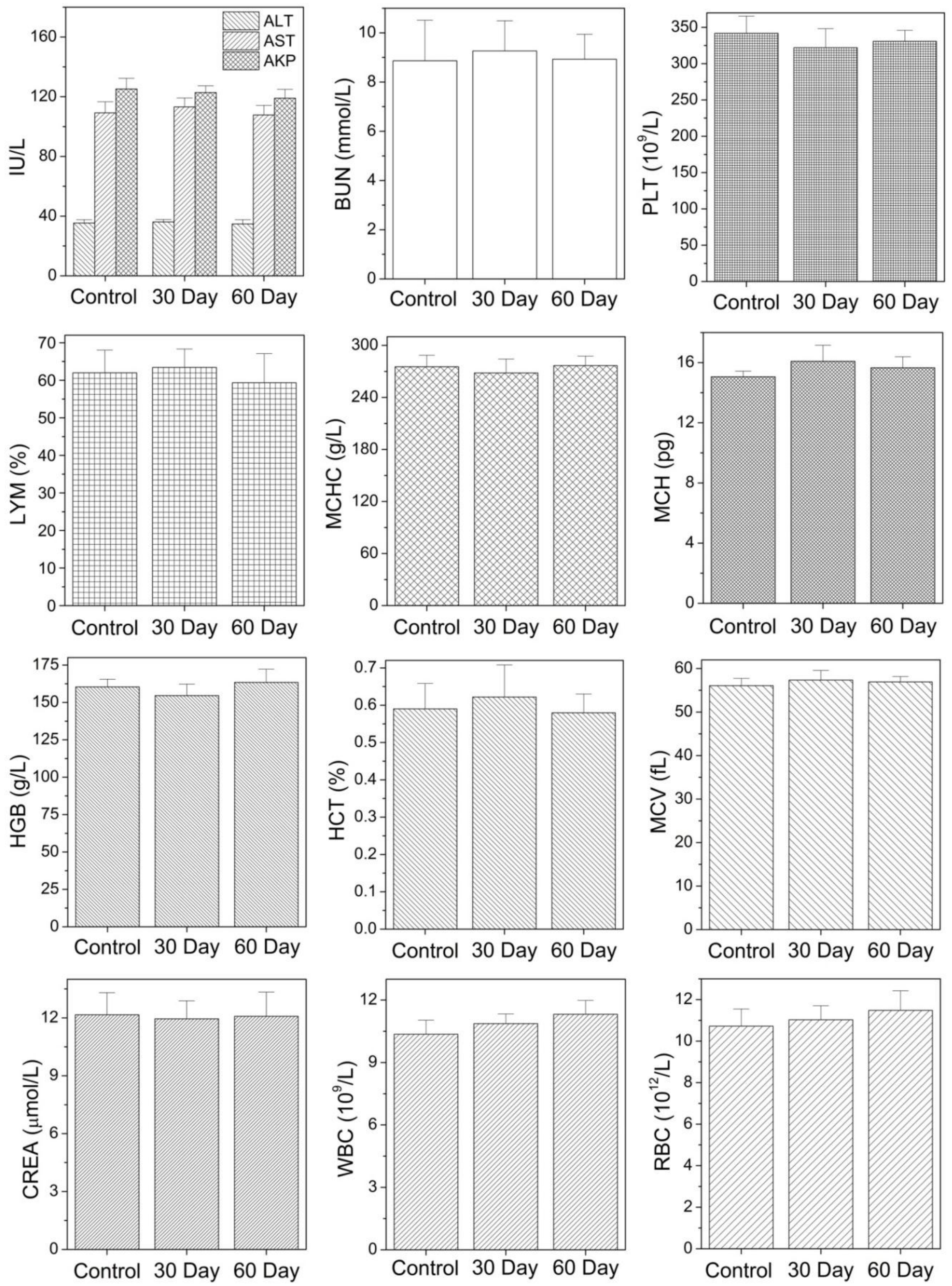

Figure S16 Main blood and biochemical indexes of nude mice at different time points after I.V. injecting MON-FA-Mn

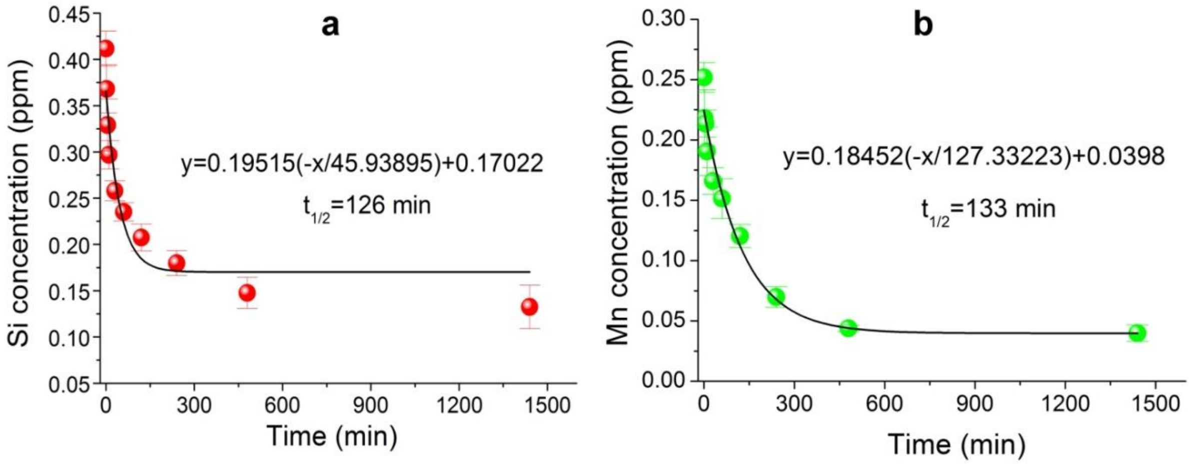

Figure S17 Blood half-lives of MON-FA-Mn obtained according to the quantification of Si and Mn atoms *via* the ICP-AES method.

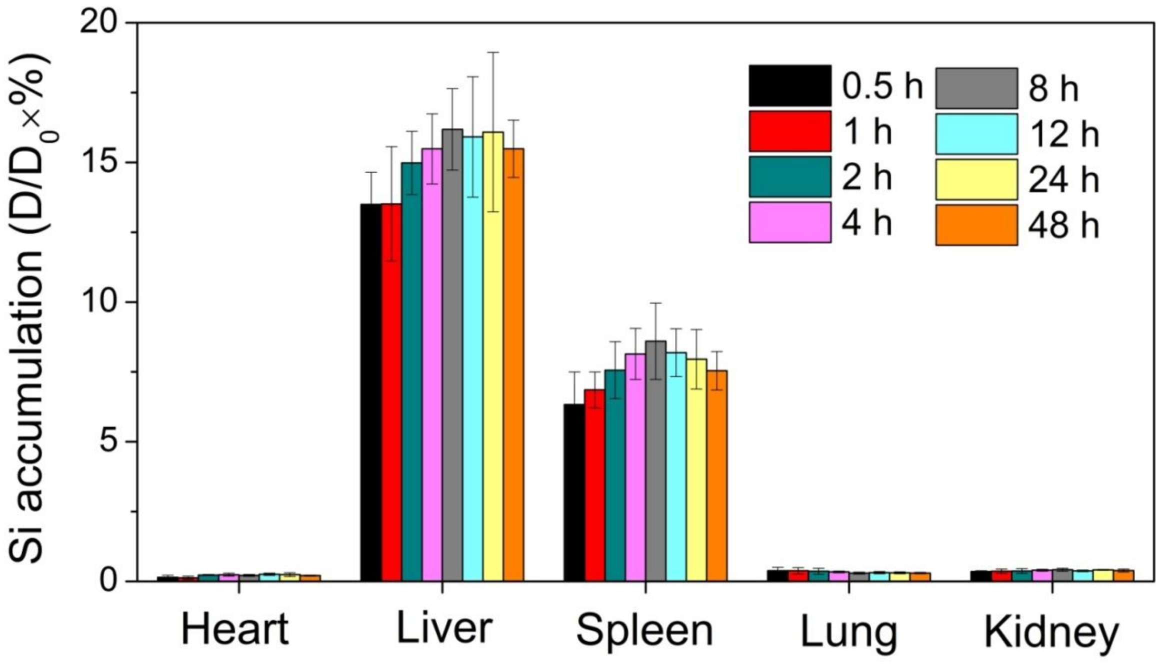

Figure S18 Time-dependent bio-distributions of Si atoms in several main organs of BLAB/c nude mice after I.V. injecting MON-FA-Mn (Mn dose: 1 mg/ Kg).

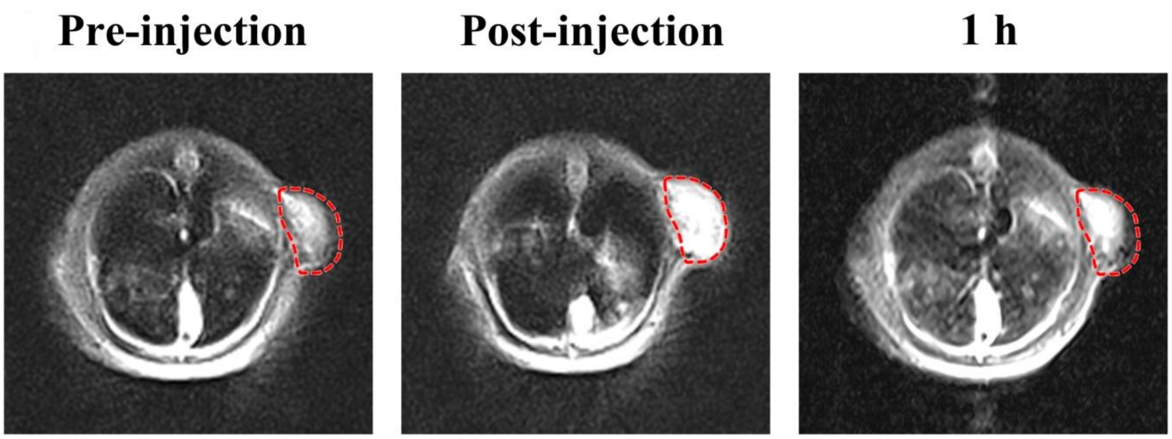

Figure S19 *In vivo* T1W MRI images of VX2 tumors subcutaneously implanted on nude mice before and after the *i.t.* injection of CLRT probe (that is, MON-FA-Mn) and 1 h post-injection (Dose: 2 mg Mn/Kg), wherein 1/T1 in the region of interest (ROI) circled by red dotted line was determined.
